# Supplementary material for: Empirical calibration of a simulation model of opioid use disorder
Source: PLoS One. 2025 Mar 27;20(3):e0310763. doi: 10.1371/journal.pone.0310763 (PMC11949371; doi:10.1371/journal.pone.0310763)
Supplement: S2 File — (DOCX) [file pone.0310763.s002.docx]

RESEARCHING EFFECTIVE STRATEGIES TO PREVENT OPIOID DEATH (RESPOND) MODEL: TECHNICAL APPENDIX

TABLE OF CONTENTS

[A. INTRODUCTION 3](#_Toc92289032)

[B. MODEL STRUCTURE 4](#_Toc92289033)

[B.1 Overview 4](#_Toc92289034)

[C.1 Key Data Sources for the Model 8](#_Toc92289036)

[C.1.1 Massachusetts Public Health Data Repository 8](#_Toc92289037)

[C.1.2 NIDA Clinical Trial Network Protocols 0051 (CTN) 8](#_Toc92289038)

[C.1.3 Medical Literature 8](#_Toc92289039)

[C.2 Components 8](#_Toc92289040)

[C.2.1 Population Dynamics 8](#_Toc92289041)

[C.2.1.1 Initial Cohort 8](#_Toc92289042)

[C.2.1.2 Aging 10](#_Toc92289043)

[C.2.1.3 Entering Cohort (New OUD Arrivals) 10](#_Toc92289044)

[C.2.2 Natural History of OUD 13](#_Toc92289045)

[C.2.3 Care Delivery 15](#_Toc92289048)

[C.2.3.1 Movement From No-Treatment to Treatment Episodes 15](#_Toc92289049)

[C.2.3.2 Treatment Initiation Effect 17](#_Toc92289050)

[C.2.3.3 Transitions Between Active and Non-Active Opioid Use While Engaged With Treatment 18](#_Toc92289051)

[C.2.3.4 Probability of Loss to Follow-Up 20](#_Toc92289052)

[C.2.4 Overdose 21](#_Toc92289053)

[C.2.4.1 Overall (non-block specific) Overdose Rates 23](#_Toc92289056)

[C.2.4.2 No Treatment 24](#_Toc92289057)

[C.2.4.2 Overdose While on Treatment 24](#_Toc92289057)

[C.2.4.3 Overdose During the Post-Treatment Period 25](#_Toc92289058)

[C.2.5 Mortality 25](#_Toc92289059)

[C.2.5.1 Fatal Overdose 25](#_Toc92289060)

[C.2.5.2 Competing risks of death (non-overdose mortality) 26](#_Toc92289061)

[C.2.5.3 Summary of the combined impact of medications for opioid use disorder on all-cause mortality in RESPOND 28](#_Toc92289062)

[I. REFERENCES 29](#_Toc92289063)

# ***A. INTRODUCTION***

The growing prevalence of Opioid Use Disorder (OUD) has resulted in an increase in opioid overdoses in the United States. Overdose is the leading cause of premature death among Americans under the age of 50 and has increased by more than 2.5 times between 1999 and 2015. Although evidence-based treatments are available for treating OUD, these treatments are under-utilized, thus the impact of opioids on the United States’ population persists.

Researchers and policy makers have made efforts to create feasible action plans for reducing the prevalence of OUD. Unfortunately, most policy makers do not have the evidence needed for informing and implementing system-level change. System-level thinking investigates how systems operate and how they can be modified to produce desired outcomes. At this time, data on system-level interventions for OUD are limited and inconsistent.

In an effort to fill the knowledge gap, simulation modeling can be used to integrate data from multiple sources to translate outcomes from clinical studies to policy-relevant data about population health and cost. By simulating state-level behaviors and practices related to OUD, we can project and evaluate the impact of relevant interventions and policies on public health outcomes and costs, hence informing practice and policy decisions to combat OUD.

The **R**esearching **E**ffective **S**trategies to **P**revent **O**pioid **D**eath (RESPOND) model is a state-transition, cohort-based model that simulates populations with high-risk opioid use in a state, including the natural history of opioid use disorder, movement on and from opioid treatment, and overdose. The model provides outputs and projections that decision-makers can use to evaluate and modify care delivery systems to match their local epidemics and available resources.

Model inputs and parameters are adaptable to users’ needs, namely, to represent heterogeneous populations, different dynamics of the drug overdose epidemic, and the effectiveness of intervention strategies in the prevention of opioid-related harms. The user, for example, can customize among other things, the demographics, time in each cycle, transition probabilities between health statuses and treatment states, and the number of health states included in the model in order to represent different structure and disease dynamics of the underlying population.

# ***B. MODEL STRUCTURE***

## ***B.1 Overview***

RESPOND is a state-transition, cohort-based [[1, 2](#Ref_2)] model that simulates the population living within a jurisdiction and who have high-risk opioid use. Typically, RESPOND simulates the population of a state, but it can also simulate a smaller area, such as a town or rural community, depending on the model parameter values. The model employs a Markov process with a weekly cycle length to accurately reflect population dynamics, clinical progression, and treatment of opioid use disorder.

The model structure comprises four main components: 1) population dynamics, 2) natural history of OUD, 3) care delivery, and 4) mortality.

The population dynamics modules simulate the epidemiology and demography of the opioid epidemic. The user can create either an open or a closed cohort simulation. In an open cohort simulation, new population “arrives” to the simulation in every time step such that the total population in the model always reflects the size of the total population with OUD living in that jurisdiction. The arrival rate represents both the development of new opioid use disorder and migration into the state among those with existing opioid use. In a closed cohort, no cohort members enter the simulation and the size of the population in the simulation dwindles over time as cohort members die.

The core simulation (*Figure 1*) of the RESPOND model involves the simulation of the natural history of opioid use disorder as a relapsing and remitting disease over a lifetime. RESPOND simulates OUD as a series of transitions between four health states of opioid use: 1) active, non-injection, 2) non-active, non-injection, 3) active injection, and 4) non-active, injection opioid use. In each time-step of the simulation, population fractions move between opioid use states. The definitions of “active” and “injection” opioid use can vary (but must be pre-specified) depending on the users’ needs and available information. In the RESPOND Massachusetts base case, “active” opioid use is defined as any reported use in the previous seven days. “Injection” opioid use reflects any injection in the preceding seven days (a person who is both injecting and using oral opioids would be categorized as “injection” in RESPOND).

The care delivery module ([*Figure 2*](#Figure_2)*)* of RESPOND simulates OUD treatment and includes four treatment types: 1) outpatient buprenorphine (Bup), 2) outpatient injectable naltrexone (Ntx), 3) outpatient methadone (Mmt) maintenance, and 4) inpatient acute drug detoxification (detox). The model is adaptable to additional intervention types to better reflect local conditions and evolutions in the treatment field. In general, treatment episodes tend to decrease movement into active drug use, increase movement into non-active drug use, and have an independent effect on overdose rates conditional on active drug use. When population disengages from a treatment and is lost to follow-up, those people enter a corresponding “post-treatment state”. The post-treatment state is a fixed interval during which relapse to active drug use is high, tolerance to opioids is lower than before treatment, and the risk of drug overdose among those actively using opioids is higher than it is in the no treatment state. The post-treatment state represents the period of vulnerability and excess overdose observed in real-world settings among patients who have recently relapsed to opioid use after a period of sustained abstinence.

The mortality module simulates both drug-related and competing risks deaths. RESPOND simulates overdose mortality by first simulating overdose incidence as a function of age and type of drug use (injection vs. non-injection use). Next, the model simulates a probability of death conditional on having had an opioid overdose. The model simulates competing causes of death through the use of standardized mortality ratios that are a function of age, sex, and type of opioid use (injection vs. non-injection).

The primary model outputs are: 1) All-cause mortality, 2) Overdose mortality, and 3) Number of people on treatment.

The simulation process is as follows: At simulation start, the model initiates a cohort of people currently living with OUD in the jurisdiction of interest. Based on data from that jurisdiction, the model assigns the current population to a drug use state, as well as a treatment block, such that the simulated population, including the prevalence of OUD treatment, reflects the status quo. Moving forward through simulated time, the sequence of simulation steps are: 1) aging of the population, 2) arrival of new population, 3) transition between OUD drug use states, 4) transitions into and out of treatment, 5) overdose, and 6) death. At the end of this sequence of processes, the model advances simulated time by one cycle (week) and repeats the process. The simulation continues until a time horizon assigned by the user.

Full model schematics are provided with the main manuscript (Figure 1 of the main manuscript).

Following sections of this document provide details of model parameters for a specific use case of the model that simulates the OUD population of the state of Massachusetts from the end of year 2012-2015 with an open cohort.


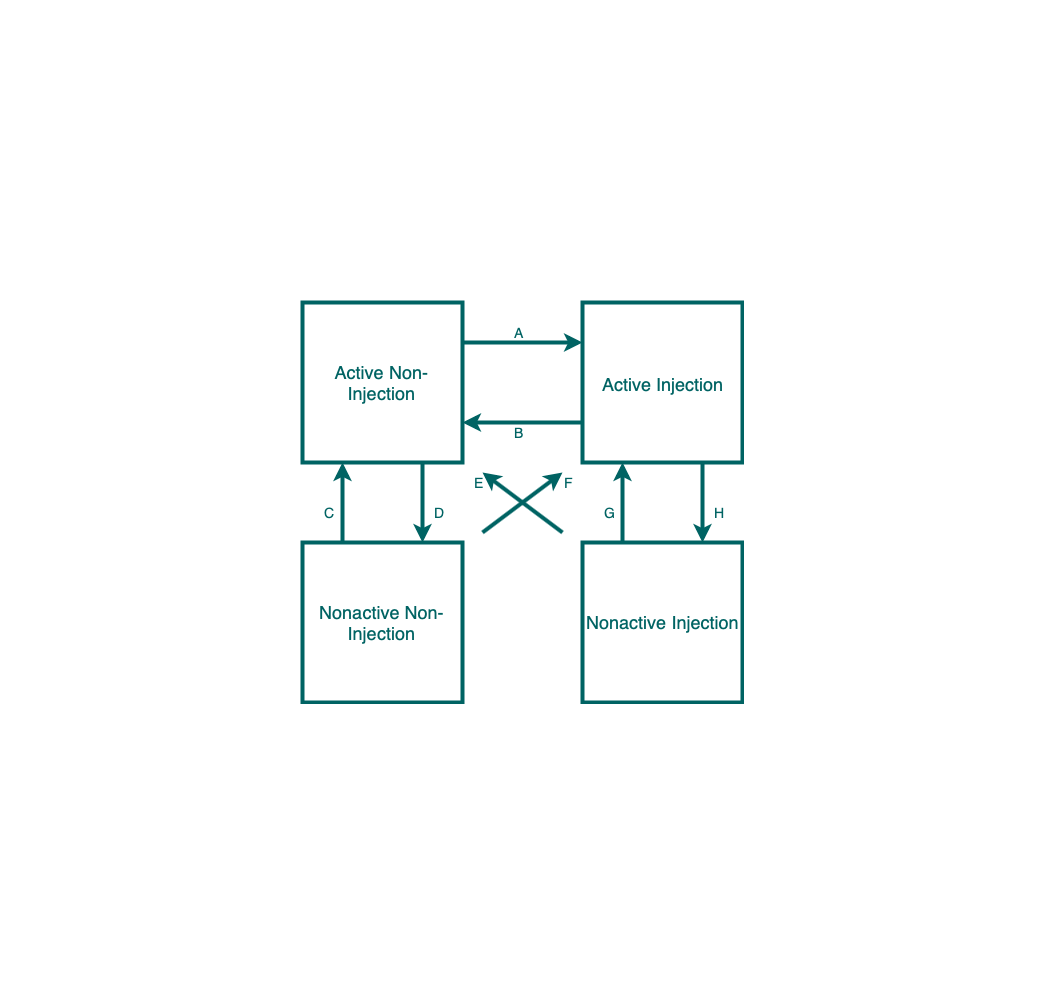


Figure 1. Core Simulation

#

Active Non-Injection

Active

Injection

Nonactive Non-Injection

Nonactive Injection

**No Treatment**

Active Non-Injection

Active

Injection

Nonactive Non-Injection

Nonactive Injection

**Detox**

Active Non-Injection

Active

Injection

Nonactive Non-Injection

Nonactive Injection

**Buprenorphine**

Active Non-Injection

Active

Injection

Nonactive Non-Injection

Nonactive Injection

**Naltrexone**

Active Non-Injection

Active

Injection

Nonactive Non-Injection

Nonactive Injection

**Methadone**

**Post-Treatment**

**Post-Treatment**

**Post-Treatment**

**Post-Treatment**

##

Figure 2. RESPOND’s care delivery module

## ***C.1 Key Data Sources for the Model***

### C.1.1 Massachusetts Public Health Data Repository

The Massachusetts Public Health Data Repository (MA PHD) is a linked longitudinal records dataset that includes administrative records and billing claims from over 15 state agencies. The spine of the database is the Massachusetts All Payers Claims database, which includes medical billing for all payers in the state. The database links person-level records from vital statistics, the Department of Corrections, Emergency Medical Services, and the Bureau of Substance Addiction Services, such that it is possible to construct longitudinal, person-level trajectories across various treatment episodes, admissions to the hospital, and overdose events [[3](#Ref_3)] RESPOND uses the MA PHD dataset to estimate parameters such as OUD epidemiology in MA and rates of transition onto treatments assuming the status quo.

### C.1.2 NIDA Clinical Trial Network Protocols 0051 (CTN)

The National Institute on Drug Abuse administers a large clinical trials network for evaluation of treatments for substance use disorders. The Clinical Trial Network (CTN) 0051 protocol was a head-to-head comparative effectiveness trial of sub-lingual buprenorphine and injectable naltrexone for individuals with opioid use disorder who were accessing acute opioid detoxification services. RESPOND uses urine toxicology data from the trial to estimate transitions between substance use states while taking buprenorphine or naltrexone, as well as health care utilization among patients with OUD [1]. [4-6]

### C.1.3 Medical Literature

In addition to the primary data sources listed above, RESPOND estimates many model parameters from the medical literature. The detailed explanation of model parameters below provides references to the relevant publications.

## ***C.2 Components***

### C.2.1 Population Dynamics

#### *C.2.1.1 Initial Cohort*

To simulate the demography and OUD epidemiology in the underlying population, RESPOND requires the initial cohort to be specified as follows: 1) age and sex distributions of people with opioid use disorder, 2) proportion of people beginning in each drug-use state, and 3) proportion of people within each treatment episode.

##### Structural Assumptions:

- No population begins the simulation in a post-treatment block.
- RESPOND does not characterize the population by race or ethnicity.

##### Methodological Notes:

[**Table 1**](#Table_1) presents the key parameters related to cohort initialization.

First, we took the estimates of the population of OUD between 2013 and 2015 from a age, sex, and county stratified capture-recapture analyses using the Massachusetts Public Health Data Warehouse (MA PHD) [7]. The capture-recapture approach provides a method to estimate the total population with high-risk opioid use in a given calendar year, including those who have not been identified as being an opioid user and do not appear in medical claims or prevalence surveys. The previous work in [[7](#Ref_7)] provides estimates for year 2012, however, as it has been noted in the paper, the data sources used before 2013 were not complete. Hence, we used data in the years with more comprehensive data sources to predict the data in 2012 which has fewer data sources.

Second, we obtained the age and sex stratified counts for alive OUD population at the end of each year by subtracting death with opioid overdose involved from the estimated total size.

Then, we applied a Negative Binomial regression on the alive counts to predict the counts in the end of 2012, with age, sex and calendar years being the covariates. An interaction of Age groups and sex was applied in the model. We treated this predicted count as the alive population size in the end of year 2012 and assumed that it constructs the initial cohort beginning in year 2013. To further identify the number of OUD in no treatment, as well as in treatment, at the beginning in year 2013, we identified OUD as we did in [[7]](#Ref_7) and then counted the number of OUD in no-treatment ($N_{0}$) in January 2013. The remaining population size is called “the number of OUD in treatment” ($T_{0}$).

**Table 1. Initializing Cohort Parameters**

| Parameter | Value | Method | Years | Stratification | Time Varying | Source |
| --- | --- | --- | --- | --- | --- | --- |
| Population size, n |  |  |  |  | Yes | MA PHD analysis update to  Barocas et al. [7] |
| Population of high-risk opioid use |  | | | | | |
| Total ($\hat{\mathbf{N}}$_OUD,t_) |  | Capture recapture | 2013-2015 | Age (3 groups^*^) & Sex | Yes | MA PHD analysis update to  Barocas et al. [[7]](#Ref_7) |
| By age-group |  | Observed | 2012-2015 | Age (18 groups^**^) & Sex | Yes | US Census 2010 |
|  | | | | | | |
| Proportion with injection drug use | 25.09% | Observed | 2013 | Age (3 groups^*^) & Sex | No | NSDUH |
| Proportion non-actively using | 9% | Estimated |  |  | No | CDC [[8]](#Ref_8)  Cedarbaum et al [[9]](#Ref_9) |
| Abbreviations:  - MA DPH: Massachusetts Department of Public Health data  - NSDUH: National Survey on Drug Use and Health  ^*^ 3 age groups: 10 – 24, 25 – 44, 45 – 99  ^**^ 18 age groups: 5-year age-groups from 10-99 | | | | | | |

####

#### *C.2.1.2 Aging*

RESPOND simulates discrete time steps (rather than continuous time) and categorical age groups or “brackets” over the lifetime. The user can define the bounds of age groups so as to match the structure of the underlying population. Aging occurs as the population progresses to the next age group after a number of cycles that is determined by the size of the age brackets. The model employs a half-cycle correction and aging occurs in discrete steps, namely only at multiples of the age group size.

##### Structural Assumptions:

- The entire population of the last age bracket (95 to 100-year-olds) is removed from the simulation at each aging cycle and replaced by the population from the previous age bracket.

#### *C.2.1.3 Entering Cohort (New OUD Arrivals)*

RESPOND can simulate either an open cohort (meaning that new individuals arrive to the population over the course of the simulation), or a closed cohort. Here we consider an open cohort simulation of OUD population in state of Massachusetts. When running the model to simulate an open cohort, arrivals to the population occur at every time step. Arrival rates are stratified by age and sex and vary over time to realistically reflect the incidence of new opioid use disorder and/or movement into the jurisdiction.

##### Structural Assumptions:

- All new populations enter the first block (“No Treatment” episode) and the first OUD state (currently active, non-injection).
- All new arrivals enter the simulation as active non-injectors under the “No Treatment” block but can transition to other feasible OUD states in the OUD transition module.

##### Methodological Notes:

***Table 2*** and ***Table 3*** present the key parameters related to entering cohort.

- The size of the entering cohort (N_enter,t_) each year defined as:

N_enter,t_ = $\hat{N}$_OUD,t_ – ($\hat{N}$_OUD,t-1_ - $\hat{N}$_FOD,t-1_ - $\hat{N}$_D_other,t-1_ ) (1)

where $\hat{N}$_OUD,t_ is the estimated OUD population in year t, $\hat{N}$_FOD,t-1_ is the estimated number of fatal overdoses, and $\hat{N}$_D_other,t_ is the estimated total number of deaths from other causes, in the previous year (t-1).

- Let $\alpha_{j,k}(t)$ be age-sex stratified weekly new OUD arrival count in year $t$, where j, and k represent age and sex strata respectively and t=2013, 2014, 2015. Then $\alpha_{j,k}\left( t \right)=\mathbf{A}\left( \mathbf{t} \right)\boldsymbol{\times}p_{j,k}\left( t \right).$ Here $\mathbf{A}\left( \mathbf{t} \right)$is the weekly non-stratified total arrivals and $p_{j,k}(t)$ denotes yearly time varying demographic proportions. We calculate $\mathbf{A(t)}$ from N_enter,t_ as $\mathbf{A}\left( \mathbf{t} \right)\boldsymbol{=}$N_enter,t_$/52$.

**Table 2. Entering Cohort Parameters**

| Parameter | Description | Value | Method | Stratification | Source |  |
| --- | --- | --- | --- | --- | --- | --- |
| N_initial_ | Initial cohort size combining no treatment size and treatment size | 215,918 | Estimated from NB regression | Age (j=1,..,3 groups^*^)  Sex (k=1,2)  Time (t=2013, 14, 15) | MA PHD Repository  Barocas et al. [[7]](#Ref_7) |  |
| N_enter,t_ | Entering cohort size | $\hat{N}$_OUD,t_ – $\hat{N}$_initial,2013_ | Calculated | Time (t=2013) |  |  |
| N_enter,t_ |  | $\hat{N}$_OUD,t_ – ($\hat{N}$_OUD,t-1_ - $\hat{N}$_FOD,t-1_ - $\hat{N}$_D_other,t-1_ ) | Calculated | Time (t=2014, 15) |  |  |
| $\hat{\mathbf{N}}$_FOD,t_ | Fatal overdoses |  | Estimated |  |  |  |
| $\hat{\mathbf{N}}$_D_other,t_ | Non-FOD deaths |  | Estimated |  | MA PHD |  |
| p_jk_(t) | Entering cohort demographic proportions at each cycle (week) |  | - Observed   Imputation of missing strata % | Age (j=1,..,5 groups^**^)  Sex (k=1,2)  Time (t=2013, 14, 15) | NSDUH MRB Statistical Inference Report [[10]](#Ref_10) |  |
| Abbreviations:  - MA DPH: Massachusetts Department of Public Health data  - NSDUH: National Survey on Drug Use and Health  - MRB: Methodological Resource Book  - NB: Negative Binomial Distribution  ^*^ 3 age groups: 10 – 24, 25 – 44, 45 – 99  ^**^ 5 age groups: 10 – 19, 20 – 24, 25 – 34, 35 – 49, 50 – 99  ^***^ Other cause mortality is from Chapter 55 assuming that is no additional death in one year. | | | | | | |

**Table 3. Entering Cohort Counts by Year**

| **Total counts**  **(population size)** | **Year** | | | |
| --- | --- | --- | --- | --- |
|  | **2012***** | **2013** | **2014** | **2015** |
| Population of OUD at time t  ($\hat{N}$_OUD,t_) | 166,835 | 226,861  (189832,301264) | 233,184  (178671,271814) | 275,070  (222160,357383) |
| Total number of non-FOD*deaths  ($\hat{N}$_D_other,t_) | 596 | 964 | 1,332 | 1,605 |
| Total number of FOD* deaths  ($\hat{N}$_FOD,t_) | 702 | 900 | 1,294 | 1,562 |
| Entering Cohort (yearly total)  (N_enter,t_) | - | **10,943** | **8,187** | **44,512** |
| Entering Cohort (Weekly total)  ($\mathbf{A}\left( \mathbf{t} \right)\boldsymbol{)}$ |  | **210** | **157** | **856** |
| *** The estimates and observed data in year 2012 do not include data from Emergency Medical Services (EMS). Subsequent years do include those data. | | | | |

### C.2.2 Natural History of OUD

RESPOND simulates opioid use as a series of transitions through four opioid use health states: 1) Non-Active and 2) Active non-injection use, as well as 3) Non-Active and 4) Active injection use ([*Figure 1*](#Figure_1)). Throughout the simulation, there is a multi-directional movement between OUD states.

Transitions between drug use compartments impact four important outcomes: 1) risk of overdose, 2) risk of death from competing causes, 3) health care utilization (cost), and 4) quality of life.

The primary sources of data for substance use transitions are studies from the medical literature.

#### Structural Assumptions:

- OUD is a remitting and relapsing process over a lifetime. There is no health state of OUD cure or permanent recovery.
- Transitions between OUD health states are not time updated.

#### Methodological Notes:

***Table 4*** presents the key parameters related to OUD transitions for no treatment.

- All Confidence Intervals (CIs) are 95%, namely calculated at α=5% level of significance.
- CIs for proportions p_E_, p_F_, p_B_, p_H_, and p_D_ are calculated using the normal approximation to binomial proportions.
- CIs for rates R_A_, R_C_, and R_D_ are provided from the manuscript and calculated assuming Poisson distribution.
- Weekly rates and proportions, calculated from the respective overall estimates, are converted to weekly transition probabilities as indicated in the “Method” column in [***Table 4***](#Table_4).

**Table 4. No-Treatment and Post-Treatment: Opioid Use Disorder Transition Parameters**

| Parameter | Description | Value | Method | Source |
| --- | --- | --- | --- | --- |
| No Treatment ($\boldsymbol{\rho}_{\boldsymbol{N}}$) | | | | |
| R_A_ | Rate of active non-injection to active injection | 4.6 per 100 PY  (3.0 , 6.6) |  | Neaigus, A., et al. [[11]](#Ref_11) |
| P_A_ | Probability of active non-injection to active injection | 0.000884  (0.000577 , 0.001268) | Calculated from R_A_:  P_A_= 1-exp{R_A_/52} |  |
| R_C_ | Rate of non-active non-injection to active non-injection | 16 per 100 PY  (12.0 , 20.5) |  |  |
| R_G_ | Rate of non-active injection to active injection |  |  |  |
| P_C_ | Probability of non-active non-injection to active non-injection | 0.00307  (0.00230 , 0.00393) | Calculated from Rate(R_C_):  P= 1-exp{R_C_/52} |  |
| P_G_ | Probability of non-active injection to active injection |  |  |  |
| p_B_ | Proportion of active injection to active non-injection | 0.34 |  | Shah, N.G., et al. [[12]](#Ref_12) |
| P_B_ | Probability of active injection to active non-injection | 0.00067  (0.00054 , 0.0008) | Calculated from p_B_:  1-exp(x)  where  x = ln(1-p_B_)/(12*52) |  |
| p_E_ | Proportion of non-active injection to active non-injection | 0.13 |  |  |
| p_F_ | Proportion of non-active non-injection to active injection |  |  |  |
| P_E_ | Probability of non-active injection to active non-injection | 0.000223  (0.000115 , 0.00034) | Calculated from p_E_:  1-exp(x)  where  x = ln(1-p_E_)/(12*52) |  |
| P_F_ | Probability of non-active non-injection to active injection |  |  |  |
| p_D_ | Proportion of active non-injection to non-active non-injection | 0.03  (0.0175 , 0.0425) |  | Nosyk, B., et al. [[13]](#Ref_13) |
| p_H_ | Proportion of active injection to non-active injection |  |  |  |
| P_D_ | Probability of active non-injection to non-active non-injection | 0.00058  (0.00032 , 0.00085) | Calculated from p_D_:  1-exp(x)  where  x = ln(1-p_D_)/(52) |  |
| P_H_ | Probability of active injection to non-active injection |  |  |  |
| Post-Treatment ($\boldsymbol{\rho}_{\boldsymbol{P}}$) | | | | |
| P_A_, P_B_, P_D_, P_E_, P_F_, P_H_, | Same estimates with no-treatment. |  |  |  |
| p^*^_C_ | Proportion of non-active non-injection to active non-injection | 0.65 | CIs are calculated using the normal approximation to binomial proportions. | Bailey et al. [[14](#Ref_14)] |
| p^*^_G_ | Proportion of non-active injection to active injection |  |  |  |
| P_C_ | Probability of non-active non-injection to active non-injection | 0.2308 | Calculated from p:  1-exp(x)  where  x = ln(1-p)/4 |  |
| P_G_ | Probability of non-active injection to active injection |  |  |  |
| - p^*^_C_ and p^*^_G_ indicate the percentage of people relapsed within a month of discharge (after inpatient detoxification). - The denominators for calculating weekly probabilities depend on whether the respective available proportion or rate estimates are yearly or monthly. | | | | |

###

### C.2.3 Care Delivery

RESPOND models OUD while engaged with treatment using the same 4-state opioid use simulation that it uses to model OUD without treatment. The 4-state OUD simulation is embedded within all treatment episodes (blocks), such that individuals may both remain engaged with treatment, but also experience periods of drug use relapse. Each treatment type has its own bi-directional transition probabilities between active and non-active use. The net movement between active and non-active use while engaged with treatment favors movement to non-active use over time.

RESPOND simulates treatment using the following parameters:

1. Probability of movement onto treatment from no treatment
2. Treatment initiation effect – the probability of ceasing active opioid use immediately after initiating treatment
3. Bi-directional movements between active and non-active opioid use while engaged with treatment
4. Probability of loss to follow-up

The population that is lost to follow-up (disengages from care) must pass through a “post-treatment period” before rejoining the simulation of OUD. The post-treatment period is a four-week time, immediately following discontinuation of a treatment, during which the risk of relapse to drug use is high, as is the risk of overdose. Population that survives the post-treatment period transitions back to the simulation of OUD without treatment.

#### *C.2.3.1 Movement From No-Treatment to Treatment Episodes*

##### Structural Assumptions:

- Only population in active opioid use states seeks OUD treatment. Population that is not currently using opioids does not seek treatment.

The main source of data to inform the probability of transition from no treatment to a treatment episode is the MA PHD.

##### Methodological Notes:

Let $\lambda_{NT}$ denotes the weekly transition rates from no-treatment to treatment. Then, weekly transition probability from no-treatment to treatment is calculated from $\lambda_{NT}$ as:

${\hat{\mathbf{P}}}_{\boldsymbol{NoTrt \to Trt . T}}$ **=** 1- exp $\left\{ {-\lambda}_{NT} \right\}$ (2)

Where $\lambda_{NT}= \frac{{\hat{\mathbf{N}}}_{\boldsymbol{Obs, NoTrt \to Trt . T}}}{{\hat{\mathbf{N}}}_{\boldsymbol{Total, NoTrt \to Trt . T}}} \times\frac{1}{4}$.

Here $\hat{N}_{Obs, NoTrt \to Trt . T}$ : the observed number of people with OUD who transitioned from no-treatment to treatment $T$in January 2013

$\hat{N}_{Total, NoTrt \to Trt . T}$ : the total number of people with OUD “*at risk*” of transitioning from no-treatment to treatment T in January 2013

The weekly transition probability $\hat{P}_{NoTrt \to Trt . T}$ is estimated using data from the MA PHD repository^[[1]](#footnote-1)^, and is stratified by age (16 groups: 5-year age-groups from 10-85, and >85years old), sex, and treatment (T= Detox, Mmt, Ntx, and Bup) (***Table 5***).

**Table 5. Weekly Transition Probabilities From No-Treatment to Treatment**

| **Age** | **Sex** | **Transition to Treatment** | | | |
| --- | --- | --- | --- | --- | --- |
|  |  | **Detox*** | **Methadone** | **Naltrexone** | **Buprenorphine** |
|  |  | ${\hat{\mathbf{P}}}_{\boldsymbol{NoTrt \to Trt . T}}\boldsymbol{=}1-e^{-\tilde{\lambda}_{ND}}$ | ${\hat{\mathbf{P}}}_{\boldsymbol{NoTrt \to Trt . T}}\boldsymbol{=}1-e^{-\lambda_{NM}}$ | ${\hat{\mathbf{P}}}_{\boldsymbol{NoTrt \to Trt . T}}\boldsymbol{=}1-e^{-\lambda_{NX}}$ | ${\hat{\mathbf{P}}}_{\boldsymbol{NoTrt \to Trt . T}}\boldsymbol{=}1-e^{-\lambda_{NB}}$ |
| 10-14 | Male | 0.0037 | 0.0037 | 0.0037 | 0.0037 |
| 10-14 | Female | 0.0064 | 0.0064 | 0.0064 | 0.0064 |
| 15-19 | Male | 0.0027 | 0.0004 | 0.0016 | 0.0023 |
| 15-19 | Female | 0.0027 | 0.0005 | 0.0016 | 0.0027 |
| 20-24 | Male | 0.0054 | 0.0009 | 0.0012 | 0.0051 |
| 20-24 | Female | 0.005 | 0.0016 | 0.001 | 0.0057 |
| 25-29 | Male | 0.0056 | 0.0016 | 0.0007 | 0.0062 |
| 25-29 | Female | 0.0042 | 0.0028 | 0.0007 | 0.0065 |
| 30-34 | Male | 0.0053 | 0.002 | 0.0006 | 0.0068 |
| 30-34 | Female | 0.0035 | 0.0027 | 0.0006 | 0.0071 |
| 35-39 | Male | 0.0049 | 0.0018 | 0.0007 | 0.0068 |
| 35-39 | Female | 0.003 | 0.0021 | 0.0008 | 0.0066 |
| 40-44 | Male | 0.0046 | 0.0017 | 0.0006 | 0.0061 |
| 40-44 | Female | 0.0027 | 0.0021 | 0.0005 | 0.0057 |
| 45-49 | Male | 0.0038 | 0.0017 | 0.0005 | 0.0052 |
| 45-49 | Female | 0.0021 | 0.0015 | 0.0005 | 0.0051 |
| 50-54 | Male | 0.0032 | 0.0012 | 0.0005 | 0.0051 |
| 50-54 | Female | 0.0016 | 0.0013 | 0.0005 | 0.0048 |
| 55-59 | Male | 0.0025 | 0.0013 | 0.0004 | 0.0048 |
| 55-59 | Female | 0.001 | 0.0013 | 0.0007 | 0.0044 |
| 60-64 | Male | 0.0018 | 0.0018 | 0.0004 | 0.0044 |
| 60-64 | Female | 0.0012 | 0.0009 | 0.0003 | 0.004 |
| 65-69 | Male | 0.0014 | 0.0014 | 0.0005 | 0.0037 |
| 65-69 | Female | 0.0011 | 0.0005 | 0.0005 | 0.0038 |
| 70-74 | Male | 0.001 | 0.001 | 0.001 | 0.0031 |
| 70-74 | Female | 0.0009 | 0.0009 | 0.0009 | 0.0045 |
| 75-79 | Male | 0.002 | 0.002 | 0.002 | 0.0039 |
| 75-79 | Female | 0.0013 | 0.0013 | 0.0013 | 0.0053 |
| 80-84 | Male | 0.0062 | 0.0031 | 0.0031 | 0.0062 |
| 80-84 | Female | 0.0026 | 0.0013 | 0.0013 | 0.0065 |
| 85-99 | Male | 0.0035 | 0.0035 | 0.0035 | 0.0069 |
| 85-99 | Female | 0.0011 | 0.0011 | 0.0011 | 0.0057 |
| * Detox transition probabilities listed here needed to be calibrated to match observed (from MA PHD) detox admissions each year from 2013-2015. We calibrated time varying multipliers $\boldsymbol{\eta}\mathbf{(}\boldsymbol{t}\mathbf{)}$on approximate rates $\tilde{\lambda}_{ND}$ to obtain accurate estimates for detox transition rates $\lambda_{ND}(t)$ where $t=2013, 2014, 2015$. In other words, $\lambda_{ND}(t)$ are calculated as $\lambda_{ND}\left( t \right)= \tilde{\lambda}_{ND}\times\boldsymbol{\eta}\mathbf{(}\boldsymbol{t}\mathbf{)}$. | | | | | |

#### *C.2.3.2 Treatment Initiation Effect*

When population begins a treatment for opioid use disorder, for example out-patient buprenorphine, a portion of the population immediately transitions from active to non-active use. Following that initial “treatment initiation effect” there is bidirectional movement between active and nonactive use states, even while engaged with treatment. The main source of data for the treatment initiation effect and for substance use transitions while engaged with buprenorphine, naltrexone, or methadone is the NIDA CTN urine toxicology data. The CTN trials collected routine periodic urine toxicology from all participants. While the published clinical trials results censored participants at the first relapse to drug use (the primary outcome of that trial), the trials continued to collect data from patients who experienced a relapse, such that the database includes longitudinal urine toxicology from patients who relapsed to active use, as well as some who remitted back to non-active use over the course of the trial. We analyzed those data in an “as treated” manner, such that RESPOND estimates realistic movements between active and non-active drug use states among people who are taking a medication. Note that relapsing to active drug use is not the same thing as loss to follow-up from treatment (see below).

##### Methodological Notes:

Upon entering treatment, a proportion of the population immediately transitions from active to non-active opioid use. This proportion $\hat{p}$_Init_Act→NonAct_ is stratified by treatment episode as follows:

- buprenorphine (Bup): 0.74, based on the proportion of observed negative (non-active) urine samples at week 1
- naltrexone (Ntx): 0.90, based on the proportion of observed negative urine samples at week 5
- methadone (Mmt): 0.57, based on the proportion of observed negative urine samples at week 5

We assume a binomial distribution and we use the Wald’s method to calculate 95% CIs for the proportion $\hat{p}$_Act→NonAct_ representing the block initiation effect.

**Table 6. Block Initiation Effects Parameters: Weekly Transition Probabilities Modeling Movement Between OUD States When Movement Between Treatment States Occurs**

| **Initial OUD state** | **Transition to** | | | |
| --- | --- | --- | --- | --- |
|  | **Treatment (**$\gamma_{T}$) | | | **Post-treatment^*^ (**$\boldsymbol{\gamma}_{\boldsymbol{P}}$**)** |
|  | **Bup** | **Ntx** | **Mmt** |  |
| Active non-injection | 0.257(0.204 , 0.309) | 0.103(0.058 , 0.148) | 0.433(0.403 , 0.462) | 1 |
| Active injection | 0.257(0.204 , 0.309) | 0.103(0.058 , 0.148) | 0.433(0.403 , 0.462) | 1 |
| Non-active non-injection | N/A** | N/A** | N/A** | N/A *** |
| Non-active injection | N/A** | N/A** | N/A** | N/A *** |
| ^*^ These estimates are the same for Bup, Ntx, Mmt, and detox.  ** There is no block initiation effect for population that is not currently using opioids, because only population that is currently using opioids seeks care in the model.  *** Calibration parameter. | | | | |

#### *C.2.3.3 Transitions Between Active and Non-Active Opioid Use While Engaged with Treatment*

We estimated Weekly OUD transition probabilities $\hat{p}$_Trt_Act→NonAct_ using Multi-State Models (MSMs)[15]. We fit separate models for each treatment: buprenorphine (Bup), naltrexone (Ntx), and methadone (Mmt), using data from the National Institute of Drug Abuse Clinical Trials Network (NIDA CTN) [4-6].

##### Structural Assumptions:

- Population engaged with treatment may move between active and non-active opioid use, but the population engaged with treatment does not change the route of administration of their opioid use. In other words, population that entered treatment using non-injection opioids will not escalate to injection drug use while still engaged with treatment (Core Simulation within OUD treatment episodes (blocks) – (*Figure 3*).

Transition probabilities between active and non-active states are the same for both injection and non-injection drug use. This structural assumption is confirmed by the MSM estimates for buprenorphine and methadone models, in which route was included as a model covariate, but was not a significant predictor of transition rates

##### Methodological Notes:

- Each MSM includes age and sex as covariates.
- Age is included as a continuous covariate in the MSM model, thus allowing estimation of the transition probabilities for age bins in which data are not available. We consider five 5 age groups: 10–19, 20–24, 25–34, 35–49, and 50–99 years old.
- OUD transition for Buprenorphine and Methadone: We keep all the weekly MSM estimates of OUD transition probabilities except week 1, which is considered as block initiation.
- OUD transition for Naltrexone: We delete the estimates for the first 4 weeks due to the inaccurate results from detoxification. Week 5 is also excluded from the analysis, as it is considered as block initiation.
- Transition probabilities from non-active to active use are defined as: $\hat{p}$_Trt_NonAct→Act_ = 1 − $\hat{p}$_Trt_Act→NonAct_


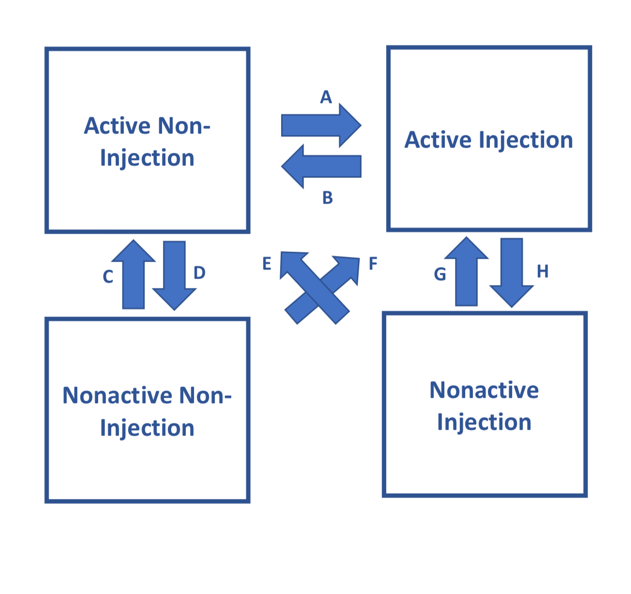


Figure 3. Core Simulation Within OUD Treatment Episodes (blocks)

**Table 7. Transition Probabilities Between Active and Non-Active Opioid Use While Engaged with Treatment**

| **Age** | **Sex** | **Initial OUD status*** | **Treatment** | | |
| --- | --- | --- | --- | --- | --- |
|  |  |  | **Buprenorphine** | **Naltrexone** | **Methadone** |
| 10-19 | Male | Active | 0.750 | 0.847 | 0.669 |
| 10-19 | Female | Active | 0.746 | 0.711 | 0.692 |
| 10-19 | Male | Nonactive | 0.156 | 0.107 | 0.112 |
| 10-19 | Female | Nonactive | 0.118 | 0.130 | 0.089 |
| 20-24 | Male | Active | 0.738 | 0.832 | 0.678 |
| 20-24 | Female | Active | 0.735 | 0.687 | 0.700 |
| 20-24 | Male | Nonactive | 0.148 | 0.099 | 0.120 |
| 20-24 | Female | Nonactive | 0.112 | 0.119 | 0.095 |
| 25-39 | Male | Active | 0.723 | 0.814 | 0.688 |
| 25-39 | Female | Active | 0.721 | 0.656 | 0.710 |
| 25-39 | Male | Nonactive | 0.139 | 0.090 | 0.128 |
| 25-39 | Female | Nonactive | 0.105 | 0.108 | 0.102 |
| 40-54 | Male | Active | 0.686 | 0.763 | 0.713 |
| 40-54 | Female | Active | 0.683 | 0.573 | 0.732 |
| 40-54 | Male | Nonactive | 0.119 | 0.072 | 0.152 |
| 40-54 | Female | Nonactive | 0.090 | 0.083 | 0.121 |
| 55-99 | Male | Active | 0.628 | 0.673 | 0.746 |
| 55-99 | Female | Active | 0.626 | 0.443 | 0.762 |
| 55-99 | Male | Nonactive | 0.096 | 0.051 | 0.192 |
| 55-99 | Female | Nonactive | 0.072 | 0.056 | 0.154 |
| ***** Probabilities listed here are transition probabilities to “Active” state. For ex:- when initial OUD status is “Active”, probabilities listed here are probabilities of staying in the “Active” state. | | | | | |

####

#### *C.2.3.4 Probability of Loss to Follow-Up*

In every time step, the population that is engaged with treatment faces a risk of disengaging from care and being lost to follow-up. Loss to follow-up differs from relapse to active drug use while remaining engaged with opioid treatment. The population that disengages with care and is lost to follow-up enters the “post-treatment state,” during which time they have a high rate of relapse to active use and a high rate of overdose among active users. The post-treatment block represents the period of time immediately following discontinuation of a medication or release from an abstinence-based setting (acute drug detoxification center, residential drug treatment, or jail), when opioid tolerance is low and the risk of overdose is higher than that of a person who never initiated treatment.

The main source of data for estimating the probability of loss to follow-up is Market Scan, a large insurance claims database containing millions of individuals who have commercial insurance coverage. As a randomized controlled trial, the CTN data cannot provide estimates of retention in care or loss to follow-up in the real world. We have previously published rates of loss to follow-up from buprenorphine and naltrexone treatment [16]. We therefore turn to Market Scan, which is nationally representative and reflects real-world practice in the U.S.

##### Structural Assumptions:

- - In RESPOND, the only way to transition into a post-treatment episode is from a corresponding treatment episode.
  - The “No Treatment” block does not have a post-treatment episode.
  - RESPOND also considers the probability of immediate relapse to active opioid use upon being lost to follow-up from treatment:

##### Methodological Notes:

The weekly transition probability from treatment to post-treatment is calculated as:

${\hat{\mathbf{P}}}_{\boldsymbol{Trt \to Post-Trt . T}}$ **=** $1-exp(-\lambda_{TP})$ (3)

where

$\lambda_{TP}$= $\frac{-log\{1-(1-p_{T})\}}{t}$ and $p_{T}$: the retention probability for treatment $T$ reported for the study time-period $t$ in literature.

***Table 8*** presents estimates of the weekly transition probabilities $\hat{P}_{Trt \to Post-Trt . T}$ based on data from Morgan et al. [16], stratified by treatment.

**Table 8. Weekly Transition Probabilities from Treatment** $\boldsymbol{T}$ **to Post-Treatment**

| **Treatment** $\boldsymbol{T}$ | $p_{T}$ | Study time-period $t$ | $\lambda_{TP}$ | ${\hat{\mathbf{P}}}_{\boldsymbol{Trt \to Post-Trt . T}}$ | **Source** |
| --- | --- | --- | --- | --- | --- |
| Bup | 0.1760 | 52 weeks | 0.0334 | 0.0328 | Morgan et al. [16] |
| Naltrexone | 0.0214 | 52 weeks | 0.0739 | 0.0713 |  |
| Methadone | 0.5240 | 20 weeks | 0.0323 | 0.0318 | Strain et al. [[17](#Ref_17)] |
| Detox | 1 | 1 week | Inf | 1 | Model assumption |

###

### C.2.4 Overdose

Every person who is actively using opioids faces the risk of overdose. The probability of overdose depends on age, sex, and route of drug use (injection vs. non-injection). The simulation has no memory of past overdose events and does not include an elevated risk of repeat overdose after experiencing a first overdose event.

#### Structural Assumptions:

- Experiencing overdose has no independent impact on current or future opioid use behaviors.
- Only the population that is in an active opioid use state faces the risk of overdose.
- The risk of overdose is different between no-treatment, treatment, and post-treatment episodes.
- The risk of overdose is lower while engaged in treatment compared to not-engaged, even among the population who are actively using drugs while engaged with treatment.

#### Methodological Notes:

***Table 9*** provides the empirically observed overdose fatalities from MA PHD.

**Table 9. Opioid Overdoses in MA**

| **Year** | **Age** | **Sex** | **Total number of people with opioid overdose** | **Number of fatal opioid overdose** | **Number of people with non-fatal overdose** | **Total overdoses** |
| --- | --- | --- | --- | --- | --- | --- |
| 2013 | 10-19 | Male | 88 | 7 | 86 | 89 |
| 2013 | 10-19 | Female | 77 | 1 | 76 | 77 |
| 2013 | 20-24 | Male | 542 | 50 | 507 | 547 |
| 2013 | 20-24 | Female | 346 | 18 | 337 | 350 |
| 2013 | 25-39 | Male | 1929 | 264 | 1746 | 1966 |
| 2013 | 25-39 | Female | 975 | 99 | 899 | 995 |
| 2013 | 40-54 | Male | 1147 | 240 | 966 | 1178 |
| 2013 | 40-54 | Female | 776 | 121 | 673 | 791 |
| 2013 | 55+ | Male | 705 | 70 | 647 | 713 |
| 2013 | 55+ | Female | 630 | 30 | 596 | 631 |
| 2014 | 10-19 | Male | 112 | 13 | 107 | 113 |
| 2014 | 10-19 | Female | 101 | 1 | 101 | 101 |
| 2014 | 20-24 | Male | 823 | 75 | 778 | 833 |
| 2014 | 20-24 | Female | 502 | 33 | 482 | 508 |
| 2014 | 25-39 | Male | 3174 | 421 | 2898 | 3268 |
| 2014 | 25-39 | Female | 1415 | 141 | 1313 | 1443 |
| 2014 | 40-54 | Male | 1607 | 314 | 1368 | 1643 |
| 2014 | 40-54 | Female | 941 | 143 | 816 | 961 |
| 2014 | 55+ | Male | 913 | 105 | 825 | 928 |
| 2014 | 55+ | Female | 754 | 48 | 704 | 756 |
| 2015 | 10-19 | Male | 114 | 16 | 109 | 116 |
| 2015 | 10-19 | Female | 95 | 3 | 93 | 96 |
| 2015 | 20-24 | Male | 800 | 97 | 742 | 827 |
| 2015 | 20-24 | Female | 465 | 37 | 446 | 478 |
| 2015 | 25-39 | Male | 3804 | 545 | 3411 | 3914 |
| 2015 | 25-39 | Female | 1650 | 157 | 1537 | 1686 |
| 2015 | 40-54 | Male | 1863 | 357 | 1594 | 1919 |
| 2015 | 40-54 | Female | 973 | 142 | 871 | 1002 |
| 2015 | 55+ | Male | 1025 | 150 | 913 | 1045 |
| 2015 | 55+ | Female | 821 | 58 | 774 | 828 |

#### *C.2.4.1* **Overall (non-block specific) overdose rates**

Yearly rate R_OD,t_ of overdose at time t for people not engaged in treatment is calculated as:

R_OD,t_ = $\frac{N_{OD,t}}{N_{OUD,t}+ \frac{1}{2} \cdot N_{enter,t}}$ × 1 PY (4)

for years t= 2013, 2014, 2015, assuming that each person contributes 1 person/year

where

N_OD,t_: number overdose cases at time t

N_OUD,t_: OUD cohort size at time t

N_enter,t_: entering cohort size at time t

Therefore, weekly overall overdose rate from anywhere in the model $o\left( t \right)= \frac{R_{OD,t}}{52}$.

***Table 10*** presents point estimates of the overdose rates by age group, sex, OUD type, and year.

**Table 10. Opioid Overdose Rates by Age, Sex, Year, and Type of OUD**

| **Age** | **Sex** | **OUD** | **Years*** | | |
| --- | --- | --- | --- | --- | --- |
|  |  |  | **2013** | **2014** | **2015** |
| 10-19 | Male | Active_Noninjection | 0.000112076 | 0.000409791 | 0.000141081 |
| 10-19 | Male | Active_Injection | 0.000674857 | 0.002467526 | 0.000849508 |
| 10-19 | Female | Active_Noninjection | 0.000162217 | 0.000458584 | 0.000417609 |
| 10-19 | Female | Active_Injection | 0.000976778 | 0.002761329 | 0.002514598 |
| 20-24 | Male | Active_Noninjection | 0.001380621 | 0.000974182 | 0.000804356 |
| 20-24 | Male | Active_Injection | 0.008313304 | 0.005865957 | 0.004843369 |
| 20-24 | Female | Active_Noninjection | 0.001557061 | 0.001189503 | 0.000777667 |
| 20-24 | Female | Active_Injection | 0.00937572 | 0.007162497 | 0.004682664 |
| 25-39 | Male | Active_Noninjection | 0.000468337 | 0.000755758 | 0.00072912 |
| 25-39 | Male | Active_Injection | 0.002820052 | 0.004550736 | 0.004390337 |
| 25-39 | Female | Active_Noninjection | 0.00037227 | 0.000421042 | 0.000529921 |
| 25-39 | Female | Active_Injection | 0.002241592 | 0.002535274 | 0.003190876 |
| 40-54 | Male | Active_Noninjection | 0.000310914 | 0.000406988 | 0.000629183 |
| 40-54 | Male | Active_Injection | 0.001872141 | 0.002450645 | 0.003788574 |
| 40-54 | Female | Active_Noninjection | 0.000304432 | 0.000306566 | 0.000557584 |
| 40-54 | Female | Active_Injection | 0.001833113 | 0.001845965 | 0.003357447 |
| 55-99 | Male | Active_Noninjection | 0.000192418 | 0.000282608 | 0.000389897 |
| 55-99 | Male | Active_Injection | 0.001158631 | 0.0017017 | 0.002347731 |
| 55-99 | Female | Active_Noninjection | 0.00020927 | 0.000261909 | 0.000126691 |
| 55-99 | Female | Active_Injection | 0.0012601 | 0.001577063 | 0.000762859 |
| * Overdose rates presented are weekly overall fixed rates $o\left( t \right)$ for each year. | | | | | |

Note that RESPOND model simulations has weekly time cycles. Let $\nu_{B}(t)$ be the overdose rate in a specific block where $B=N, T$ or $P$ for blocks no-treatment, treatment, and post-treatment. Then, weekly overdose probabilities P_OD,B,t_ are calculated from the respective overdose rates as:

P_OD,B,t_ = 1 − $e^{-\nu_{B}(t)}$ (5)

where $B=N, T$ or $P$.

#### *C.2.4.2 No Treatment*

Weekly overdose rates in no treatment $\nu_{N}(t)$ were calculated by applying a multiplier $\mathbf{m}_{\mathbf{N}}\boldsymbol{\in}\mathbb{R}^{\mathbf{+}}$ on overall overdose rates $o\left( t \right)$ as $\nu_{N}(t)= o(t)\times\mathbf{m}_{\mathbf{N}}$. There were no data available to inform the rate multiplier $\mathbf{m}_{\mathbf{N}}$. Therefore, we decided to calibrate overdose rate multiplier of no treatment block.

#### *C.2.4.3 Overdose While on Treatment*

The risk of overdose for people engaged in treatment, is derived by applying a multiplier parameter $\mathbf{m}_{\mathbf{T}}$ to the respective no-treatment $\nu_{N}(t)$ estimates. i.e., the weekly overdose rate at time t for treatment $T$ is:

$\nu_{T}(t)=\nu_{N}(t) \times\mathbf{m}_{\mathbf{T}}$ (6)

where $\nu_{N}\left( t \right)$ is the no-treatment overdose rate at time t, and $\mathbf{m}_{\mathbf{T}}$ $\boldsymbol{\in}(0, 1)$..

**Table 11. Multipliers of Overdose Rates by Treatment** $T$

| **Treatment** $T$***** | $\mathbf{m}_{\mathbf{T}}$ | **95% CI** | **Source** |
| --- | --- | --- | --- |
| Buprenorphine | 0.405 | [0.35 , 0.46] | Morgan et al. [[18]](#Ref_18) |
| Naltrexone | 0.864 | [0.42, 1.31] injectable |  |
| Methadone | 0.752** | Non-parametric uncertainty distribution from bootstrapping of data | Morgan et al. [[18]](#Ref_18)  Sordo et al. [[19](#Ref_19)] |
| ***** We assume no overdoses occur in the detox block. Therefore, overdose rate is zero.  ****** $\mathbf{m}_{\mathbf{Meth}}\boldsymbol{=}\mathbf{m}_{\mathbf{Bup}} \boldsymbol{\times}\frac{\boldsymbol{Meth rate}}{\boldsymbol{Bup rate}}\boldsymbol{=}0.405\boldsymbol{\times}\frac{2.6}{1.4}$ | | | |

#### *C.2.4.4 Overdose During the Post-Treatment Period*

During the post-treatment period, individuals face a risk of overdose higher than that of people who never initiated a treatment. Therefore, we model post-treatment overdose rates $\nu_{P}(t)$ with a multiplier $\mathbf{m}_{\mathbf{P}}$ (greater than 1) applied on no-treatment overdose rates such that $\nu_{P}(t)= \nu_{N}(t)\times\mathbf{m}_{\mathbf{P}}$. There were no data available to inform post-treatment overdose rate multiplier $\mathbf{m}_{\mathbf{P}}$. Therefore, we decided to calibrate overdose rate multiplier of post-treatments.

### C.2.5 Mortality

RESPOND simulates mortality through two independent mechanisms, fatal opioid overdose and non-overdose death.

#### *C.2.5.1 Fatal Overdose*

The population that experiences overdose then faces a probability of death conditional on having had an overdose. This conditional probability of death, given an opioid overdose, is generalizable to all overdose cases and is therefore not stratified by age, sex, or OUD status. The population that survives an overdose does not change substance use as a result of the overdose. The probability of death conditional on having experienced an overdose is a time updated variable, reflecting changes to drug supply over time. Adjusting the conditional probability of overdose death provides a mechanism to reflect the growing penetration of fentanyl in local drug supplies, which is a major dynamic underlying mounting overdose deaths in the U.S.

The probability $\mathbf{f(t)}$ of fatal overdose at year t is calculated as:

$\mathbf{f(t)}$ = $\frac{N_{FOD,t}}{N_{OD,t}}$ (7)

where N_FOD,t_ is the total number of fatal overdoses, and N_OD,t_ is the total number of all-type overdoses.

| **Table 12. Probabilities of fatal overdose.** | |
| --- | --- |
| **Year t** | $\mathbf{f(t)}$ |
| 2013 | 0.1248 (0.1161 , 0.1338)* |
| 2014 | 0.1251 (0.1179 , 0.1324)* |
| 2015 | 0.1346 (0.1275 , 0.1417)* |
| * Empiricaly calculated 95% CI using the Poisson distribution assumption for both counts in numerator and denominator of Eq. ( 7 ) | |

# *C.2.5.2 Competing risks of death (non-overdose mortality)*

Competing risks mortality includes deaths from conditions such as infectious endocarditis and sepsis, as well as medical comorbidities that accrue over a lifetime. The general approach to estimating competing risks of death is to apply standardized mortality ratios (SMRs) reflecting elevated mortality among drug users to age-sex stratified actuarial lifetables for the U.S. {Zeger, 1986 #24}

***Table 13*** presents weekly death probabilities $P_{NOD}$ calculated from lifetables in National Vital Statistic System (NVSS) [[20](#Ref_20)] after excluding overdoses.

| **Table 13. Overdose excluded weekly death probabilities derived from lifetables** | | |
| --- | --- | --- |
| **Age** | **Sex** | **Probability of Death** |
| 10-14 | Male | 3.23e-06 |
| 10-14 | Female | 2.33e-06 |
| 15-19 | Male | 1.22e-05 |
| 15-19 | Female | 5.32e-06 |
| 20-24 | Male | 2.20e-05 |
| 20-24 | Female | 7.85e-06 |
| 25-29 | Male | 2.43e-05 |
| 25-29 | Female | 1.00e-05 |
| 30-34 | Male | 2.77e-05 |
| 30-34 | Female | 1.41e-05 |
| 35-39 | Male | 3.37e-05 |
| 35-39 | Female | 1.90e-05 |
| 40-44 | Male | 4.50e-05 |
| 40-44 | Female | 2.83e-05 |
| 45-49 | Male | 6.83e-05 |
| 45-49 | Female | 4.41e-05 |
| 50-54 | Male | 0.000113101 |
| 50-54 | Female | 7.06e-05 |
| 55-59 | Male | 0.000173492 |
| 55-59 | Female | 0.000105002 |
| 60-64 | Male | 0.000253182 |
| 60-64 | Female | 0.000149513 |
| 65-69 | Male | 0.000350328 |
| 65-69 | Female | 0.000223448 |
| 70-74 | Male | 0.000535512 |
| 70-74 | Female | 0.000363913 |
| 75-79 | Male | 0.00084698 |
| 75-79 | Female | 0.000601651 |
| 80-84 | Male | 0.001427294 |
| 80-84 | Female | 0.001055789 |
| 85-89 | Male | 0.002907422 |
| 85-89 | Female | 0.002393584 |
| 90-94 | Male | 0.002907422 |
| 90-94 | Female | 0.002393584 |
| 95-99 | Male | 0.002907422 |
| 95-99 | Female | 0.002393584 |

Weekly non-overdose death rates $R_{NOD}$ are calculated as

$R_{NOD}=\ln\left( 1-P_{NOD} \right)\times SMR$ ( 8 )

where SMR is calculated as:

SMR = $\frac{N_{D\_other}}{R_{D} \times N_{\mathrm{OUD}}}$ (9)

where N_D_other_ is the number of observed deaths not due to opioid overdose in 2013 from NVSS, R_D_ is the 2010 census death rate, and N_OUD_ is the size of the OUD population based on chapter 55 estimation.

We construct CIs around the SMRs estimates assuming that the N_D_other_ follows a Poisson distribution and using the normal approximation (***Table 14***).

| **Table 14. Standardized Mortality Rates (SMRs)** | | |
| --- | --- | --- |
| **sex** | **OUD type** | **SMRs**  **(95% CIs)** |
| Male | Active - Non-injection | 1.79(1.58 , 2.00) |
| Male | Active - Injection | 4.41(3.84 , 4.98) |
| Male | Non-active – Non-injection | 1.83(1.15 , 2.50) |
| Male | Non-active - Injection | 4.59(2.71 , 6.46) |
| Female | Active – Non-injection | 2.31(1.99 , 2.63) |
| Female | Active - Injection | 5.67(4.81 , 6.53) |
| Female | Non-active – Non-injection | 2.30(1.29 , 3.31) |
| Female | Non-active - Injection | 5.62(2.87 , 8.37) |

Finally, we convert weekly non-overdose death rates to probabilities as 1 − $e^{-R_{NOD}}$.

# *C.2.5.3 Summary of the combined impact of medications for opioid use disorder on all-cause mortality in RESPOND*

Medications for opioid use disorder (MOUD) have two independent effects on mortality that combine to provide synergies in the simulation:

- - - 1. The population that is engaged with MOUD treatment experiences a net movement toward non-active drug use. Because there is no risk of overdose while not using drugs, MOUD tend to decrease the rate of overdose in the population. In addition, movement out of active drug use states reduces exposure to the high standardized mortality ratios (SMRs) of active drug use and thereby reduce non-overdose mortality as well.
      2. Among those who are actively using drugs when taking an MOUD, the MOUD has an independent effect on overdose risk, such that even those who are using have lower risk of death than those who are using drugs while not engaged with MOUD treatment

# ***I. REFERENCES***

1. Siebert, U., et al., *State-Transition Modeling: A Report of the ISPOR-SMDM Modeling Good Research Practices Task Force-3.* Value in Health, 2012. **15**(6): p. 812-820.
2. Sonnenberg, F.A. and J.R. Beck, *Markov-Models in Medical Decision-Making - a Practical Guide.* Medical Decision Making, 1993. **13**(4): p. 322-338.
3. *Massachusetts Public Health Data Warehouse*. 2020 [cited 2020 March 7]; Available from: <https://www.mass.gov/public-health-data-warehouse-phd>.
4. Lee, J.D., et al., *Comparative effectiveness of extended-release naltrexone versus buprenorphine-naloxone for opioid relapse prevention (X:BOT): a multicentre, open-label, randomised controlled trial.* Lancet, 2018. **391**(10118): p. 309-318.
5. Lee, J.D., et al., *NIDA Clinical Trials Network CTN-0051, Extended-Release Naltrexone vs. Buprenorphine for Opioid Treatment (X:BOT): Study design and rationale.* Contemp Clin Trials, 2016. **50**: p. 253-64.
6. Nunes, E.V., et al., *Ethical and clinical safety considerations in the design of an effectiveness trial: A comparison of buprenorphine versus naltrexone treatment for opioid dependence.* Contemporary clinical trials, 2016. **51**: p. 34-43.
7. Barocas, J.A., et al., *Estimated Prevalence of Opioid Use Disorder in Massachusetts, 2011-2015: A Capture-Recapture Analysis.* American Journal of Public Health, 2018. **108**(12): p. 1675-1681.
8. Centers for Disease Control and Prevention. *HIV Infection Risk, Prevention, and Testing Behaviors among Persons Who Inject Drugs—National HIV Behavioral Surveillance: Injection Drug Use, 23 U.S. Cities, 2018. HIV Surveillance Special Report 24*. [http://www.cdc.gov/hiv/library/reports/hivsurveillance.html. Published February 2020](http://www.cdc.gov/hiv/library/reports/hivsurveillance.html.%20Published%20February%202020).
9. Cedarbaum, E.R. and Banta-Green, C.J. *Health behaviors of young adult heroin injectors in the Seattle area*. Drug Alcohol Depend, 2016. **158**: p. 102-109.
10. Substance Abuse and Mental Health Services Administration, R., MD, *2015 National Survey on Drug Use and Health: Methodological Resource Book* Center for Behavioral Health Statistics and Quality 2017(Section 13, Statistical Inference Report).
11. Neaigus, A., et al., *Transitions to Injecting Drug Use Among Noninjecting Heroin Users: Social Network Influence and Individual Susceptibility.* JAIDS Journal of Acquired Immune Deficiency Syndromes, 2006. **41**(4): p. 493-503.
12. Shah, N.G., et al., *Longitudinal predictors of injection cessation and subsequent relapse among a cohort of injection drug users in Baltimore, MD, 1988-2000.* Drug Alcohol Depend, 2006. **83**(2): p. 147-56.
13. Nosyk, B., et al., *Characterizing longitudinal health state transitions among heroin, cocaine, and methamphetamine users.* Drug Alcohol Depend, 2014. **140**: p. 69-77.
14. Bailey, G.L., D.S. Herman, and M.D. Stein, *Perceived relapse risk and desire for medication assisted treatment among persons seeking inpatient opiate detoxification.* J Subst Abuse Treat, 2013. **45**(3): p. 302-5.
15. Jackson, C., *Multi-State Models for Panel Data: The msm Package for R.* 2011, 2011. **38**(8): p. 28.
16. Morgan, J.R., et al., *Injectable naltrexone, oral naltrexone, and buprenorphine utilization and discontinuation among individuals treated for opioid use disorder in a United States commercially insured population.* J Subst Abuse Treat, 2018. **85**: p. 90-96.
17. Strain, E.C., et al. *Dose-response effects of methadone in the treatment of opioid dependence.* Annals of Internal Medicine, 1993. **119**(1): p. 23-27.
18. Morgan, J.R., et al., *Overdose following initiation of naltrexone and buprenorphine medication treatment for opioid use disorder in a United States commercially insured cohort.* Drug and Alcohol Dependence, 2019. **200**: p. 34-39.
19. Sordo, L., et al. *Mortality risk during and after opioid substitution treatment: systematic review and meta-analysis of cohort studies.* BMJ, 2017. **357**: p. j1550.
20. National Center for Health Statistics: National Vital Statistics System *Public Use Data File Documentation Mortality Multiple Cause-of-Death*. Centers for Disease Control and Prevention, 2013. <https://www.cdc.gov/nchs/nvss/mortality_public_use_data.htm>

1. DPH Chapter 55 Data warehouse, datasets used include: APCD, BSAS, CASEMIX, DEATH, BIRTH, MATRIS, and PMPD [↑](#footnote-ref-1)
