# Supplementary material for: Empirical calibration of a simulation model of opioid use disorder
Source: PLoS One. 2025 Mar 27;20(3):e0310763. doi: 10.1371/journal.pone.0310763 (PMC11949371; doi:10.1371/journal.pone.0310763)
Supplement: S3 File — (DOCX) [file pone.0310763.s003.docx]

**Empirical calibration of a simulation model of opioid use disorder**

R. W. M. A. Madushani^1^, PhD, Jianing Wang^2^, MSc, Michelle Weitz^1^, MS, Benjamin P. Linas^1,3^, MD, Laura F. White^2^, PhD, Stavroula A. Chrysanthopoulou^4^, PhD

**List of Figures**


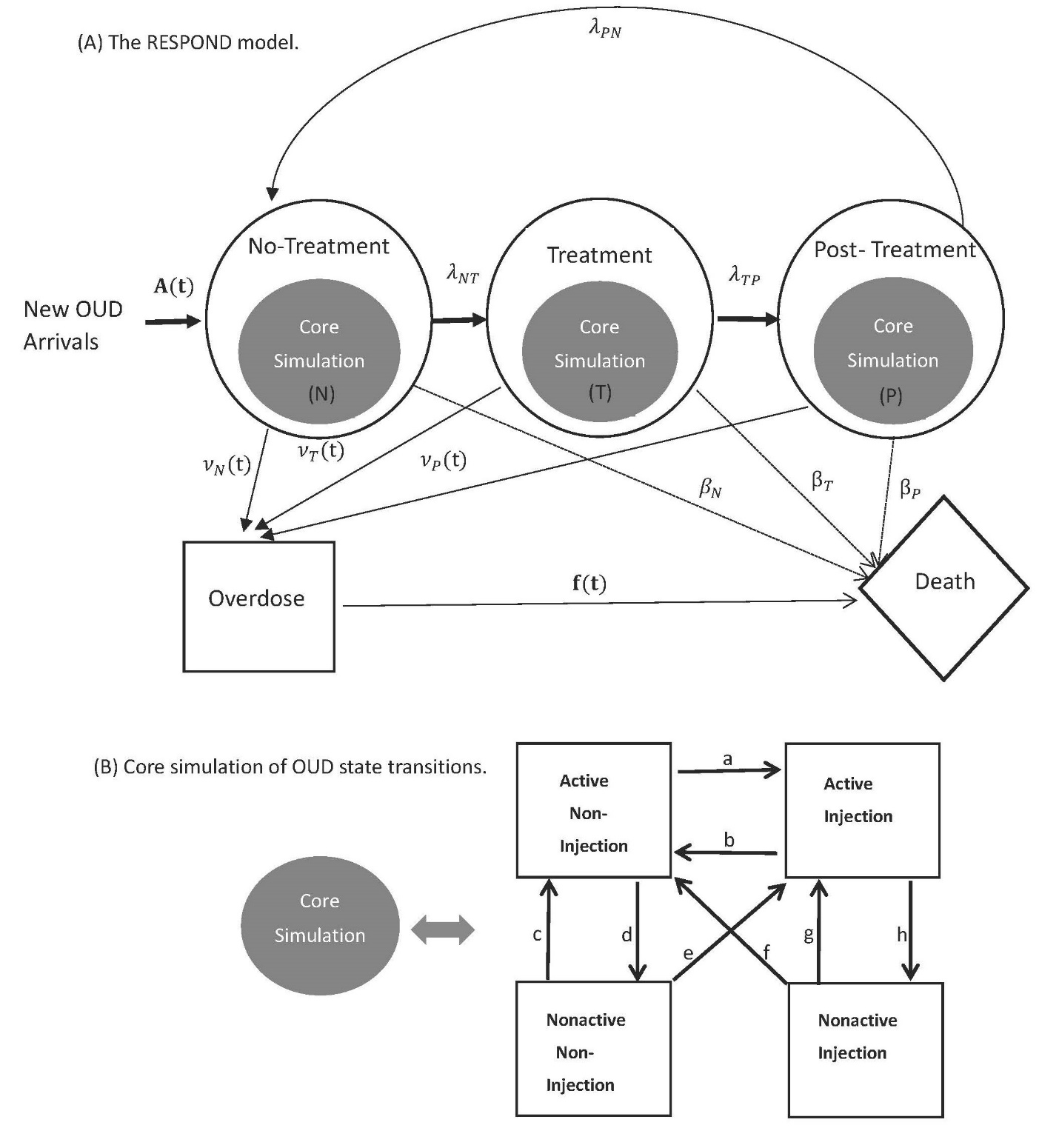


**Figure 1.** Model Structure of the RESPOND Model. (A) Overall model structure consists of three main health states: No-treatment, Treatment and Post-treatment denoted by $N, T$ and $P$ respectively. Both $T$ and $P$ are stratified by four treatment types: buprenorphine, naltrexone, methadone, and detox. $\mathbf{A(t)}$ denotes yearly time varying total new arrivals, and $\lambda$ denotes the transition rates between health states N, T and P. $\nu(t)$ denotes yearly time varying overdose rates parameter in health states, and $\mathbf{f(t)}$ denotes yearly time varying fatal overdose proportion applied on all overdoses (fatal and non-fatal combined) resulted from all health states. $\beta$ denotes non-overdose related other cause mortality rates in health states N, T and P represented by dashed arrows. (B) Core simulation of substance use state transitions within each health state. Eight different transition probabilities in the core simulation are denoted by $a, b, c,d,e,f,g$ and $h$.





**Figure 2.** Flow diagram of the empirical calibration algorithm.


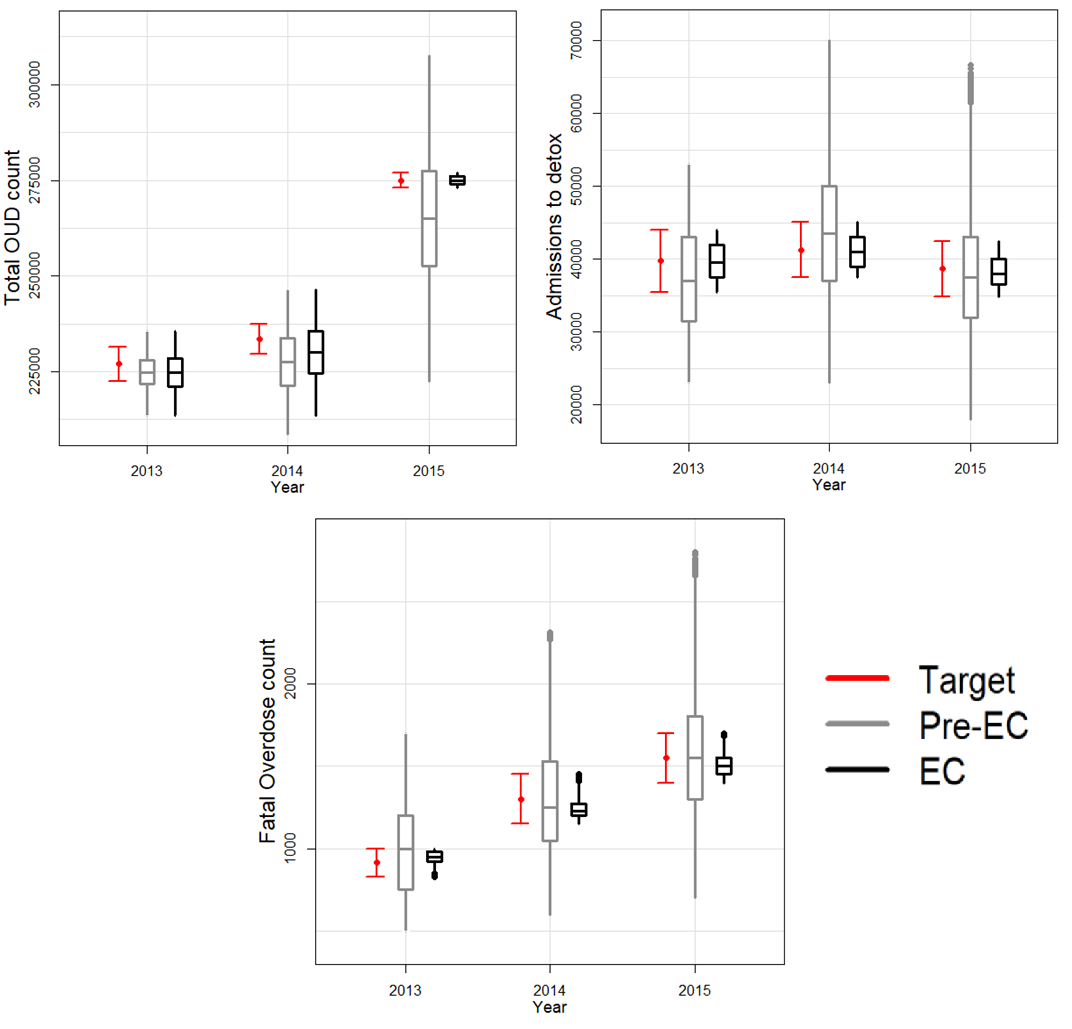


**Figure 3.** Calibration results: Box-plots presenting the distributions of actual versus resulting (simulated) calibration targets (“Total OUD counts”, “Admissions to Detox”, “Fatal Overdose Counts”) from each version of the calibrated RESPOND model over time.

The red boxplots (“Target”) present the actual distributions of the calibration targets based on point estimates and 95% CIs from the literature.

The grey boxplots (“Pre-EC”) present the distributions of the calibration targets based on RESPOND simulations when using values for the model parameters randomly selected from the respective plausible ranges which determine the marginals of the multidimensional parameter space for implementing the LHS sampling design, prior to the Empirical Calibration (EC) process. As expected, there is a large variability in the respective distributions of the simulated calibration targets.

The black boxplots (“Pre-EC”) present the distributions of the calibration targets based on RESPOND simulations when using accepted values for the model parameters resulted from the Empirical Calibration (“EC”) process.


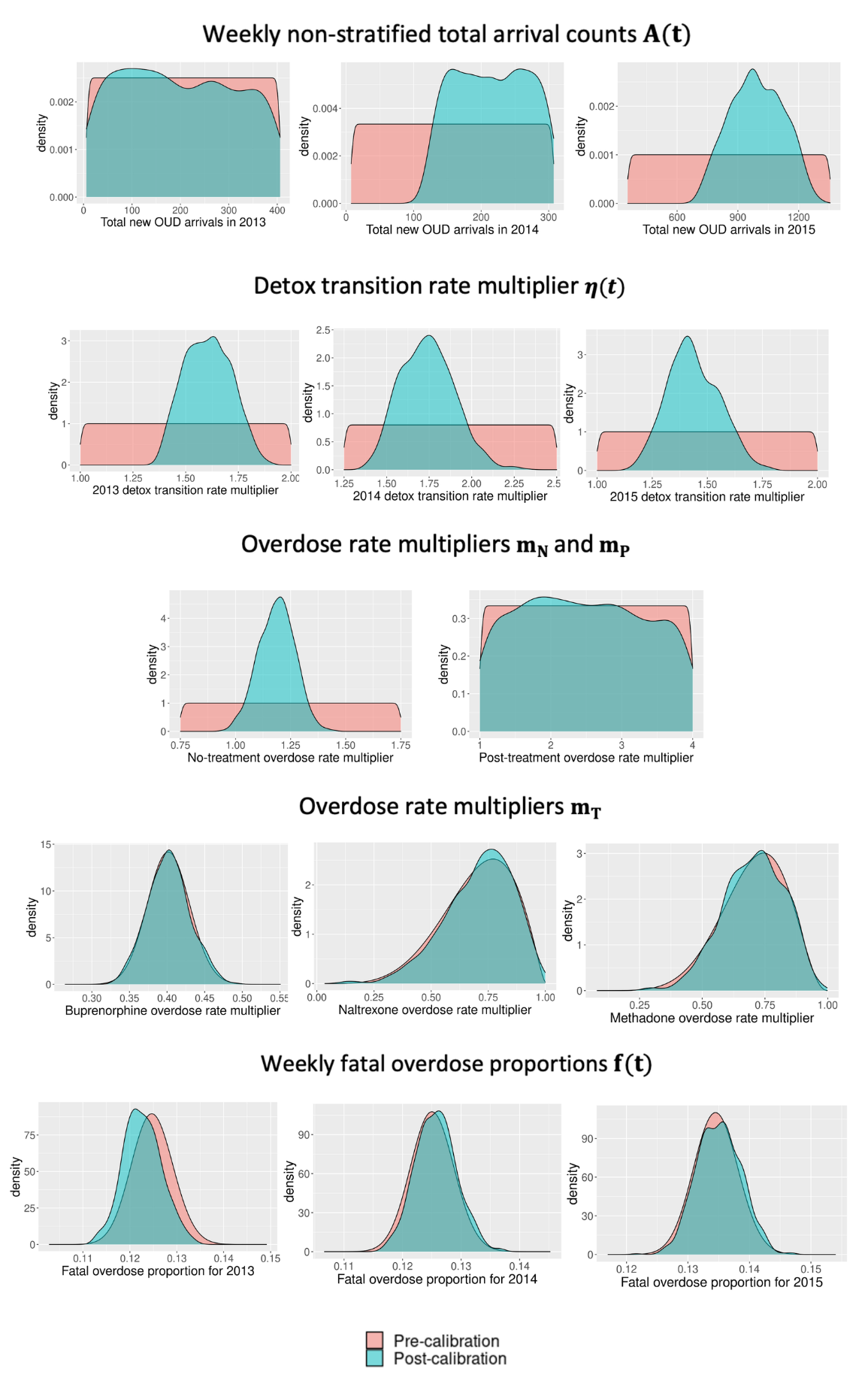


**Figure 4.** Distribution of values for the calibrated parameters of the RESPOND model. Results from the pre- and post- empirical calibration process.

The blue density plots (“Pre-calibration”) correspond to the marginal distributions specified by the plausible values for the calibrated RESPOND parameters which determine the multidimensional parameter space for implementing the LHS sampling design, prior to the Empirical Calibration (EC) process.

The pink density plots (“Post-calibration”) correspond to the marginal distributions specified by the accepted values of the calibrated RESPOND parameters resulting in from the Empirical Calibration (EC) process.

Marginal distributions of remaining parameters (substance use transitions): $\boldsymbol{\gamma}_{\boldsymbol{P}}$, $\boldsymbol{\rho}_{\boldsymbol{N}}$ and $\boldsymbol{\rho}_{\boldsymbol{P}}$ are shown in Figure S4.


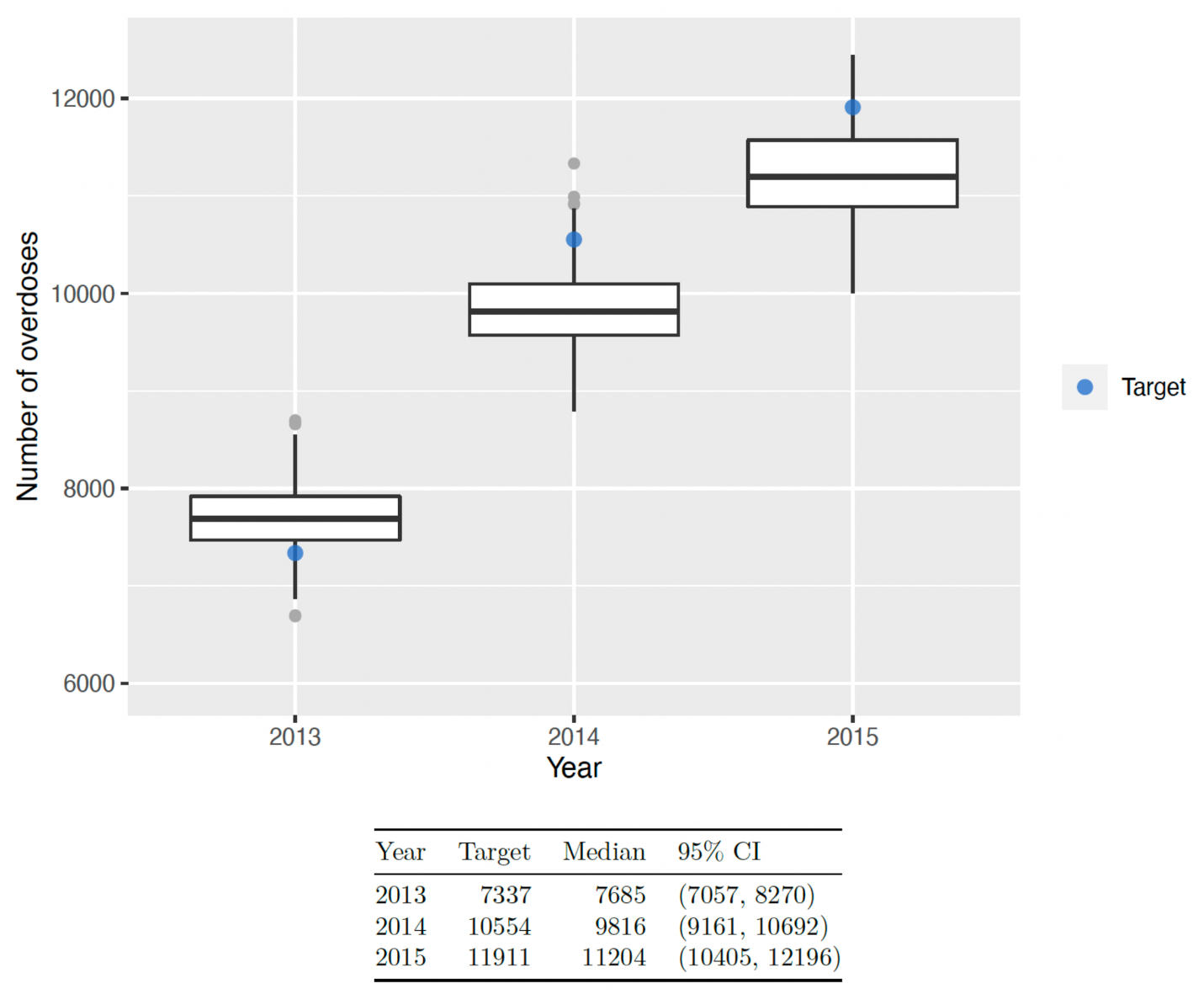


**Figure 5.** Comparison of year-end all overdose counts (non-fatal and fatal combined) model outcomes to observed overdose count targets from MA PHD.


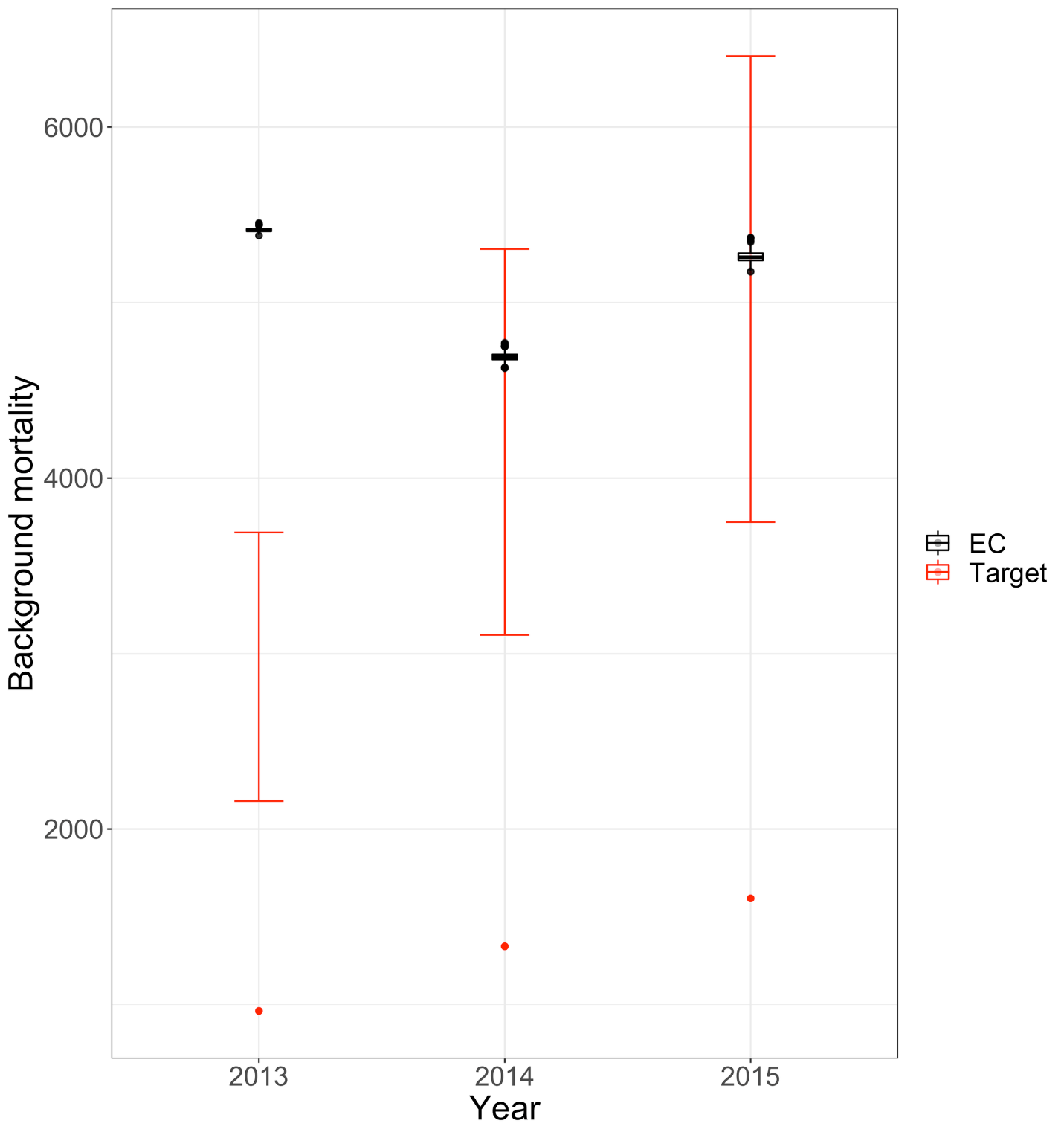


**Figure 6.** Other cause mortality model outcomes in comparison to target death counts that are corrected to be between 2.4-4.1 times the size of overdose deaths. Label EC represents death counts due to competing risks resulted from the empirically calibrated model. Red dots and red error bars represent the observed counts from MA PHD and the corrected ranges respectively.





**Figure 7.** Active vs non-active OUD percentages resulted from the calibrated model. (A) Overall active vs non-active percentages across all health states each year. (B) Yearly percentages of active vs non-active users in different health states.
