# Supplementary material for: Empirical calibration of a simulation model of opioid use disorder
Source: PLoS One. 2025 Mar 27;20(3):e0310763. doi: 10.1371/journal.pone.0310763 (PMC11949371; doi:10.1371/journal.pone.0310763)
Supplement: Attachment — Submitted filename: PLOS1_Manuscript_PONE-D-24-12466_Response to Reviewers.pdf [file pone.0310763.s005.pdf]

PLOS1 Revised Manuscript [PONE-D-24-12466]

Title: Empirical calibration of a simulation model of opioid use disorder

Authors: R. W. M. A. Madushani[1], PhD, Jianing Wang[2], MSc, Michelle Weitz[1], MS, Benjamin P. Linas[1,3], MD, Laura F. White[2], PhD, Stavroula A. Chrysanthopoulou[4], PhD

Response to Reviewers

Contents

Response to Reviewers 1

INTRODUCTION 2

Reviewer 1 2

Reviewer 2 6

INTRODUCTION

In this letter we provide a detailed, point-by-point response to all comments we received from the Reviewers.

Reviewer 1

In this article a dynamic model that simulates OUD population in Massachusetts is developed. The model is calibrated using 3 targets related to OUD data. The model includes different health and treatment statutes. One of the aims of the outcome of this study is to provide valuable information to health institutions.

The topic of the article is very important and worthy of study. The calibration of the model to target data is complex since there are many uncertain parameters in the model. The authors developed a plausible method to estimate the parameters. Although as the authors mentioned there are some non-identifiable parameters that can affect the outcomes of this study. The authors mentioned some limitations of this study which is important.

The article can be improved in many ways so readers can grasp the main ideas of the paper without reading it several times.

These are some of the aspects that should be addressed.

Comment 1.

The presentation of Figure 1 should be improved. Adding the parameters to the diagram would help the readers to understand the model in an easier way. For instance, the Lambdas.

Response 1.

We appreciate this feedback. We have revised Figure 1 accordingly.

Comment 2.

Maybe I missed but explain why there is no transitions between nonactive no-injection and nonactive injection.

Response 2.

In order to go from "non-injection" → "injection" ("injection" → "non-injection") a person needs to first pass through the respective "active" drug use state.

Comment 3.

The 8 different transition probabilities in the core simulation (a, b, c, d, e, f, g and h) should be included in one of the Figures so readers can grasp better the model and dynamics.

Response 3.

Thank you. We have revised Figure 1 accordingly.

Comment 4.

The presentation of Figure 3 should be improved. It is difficult to differentiate between target, EC and Pre-EC.

Response 4.

Thank you for the comment. We have taken the following actions to improve this figure:

1) Expand the caption elaborating on the details of the data depicted, so as to make it easier to understand the information presented in this Figure.
2) Presenting the 3 boxplots at each time point side-by-side instead of one on top of the others.

Comment 5.

Please clarify about the two sources of mortality in RESPOND model. Please make it clear that there is no overlap between these two types of deaths. Does the real data provide nonoverlap mortality data?

Response 5.

We added a relevant note in the text

Comment 6.

A better explanation can be given to the cohort initiation. Especially the last sentence

"The balance of arrivals and deaths results in the total Massachusetts OUD population size"

Response 6.

We have revised the manuscript accordingly.

Comment 7.

The model is very difficult to calibrate since there are some functions that need to be calibrated. Section 2.1. It is not clear the type of model. Please add a paragraph about the type of model. The process shown in Figure 1 can be achieved with different type of models.

Response 7.

We have revised the manuscript accordingly.

Comment 8.

Figure 1. It is not clear why the transition to the death state occurs at the same rate beta for individuals in no-treatment, treatment and post-treatment. It seems that should be different. This requires a good explanation in the manuscript.

Response 8.

Thank you for your comment. As shown in Figure 1 there are different risks of overdose from different states (No-trt, trt, or post trt). Then the risk of an overdose to be fatal is f(t). There is also a different risk of death from other causes depending on the state.

Comment 9.

Table 1. Please add more details about how the year OUD count is done. Please show the values of the 3 sources of OUD count. No overlap between them?

Response 9.

Details about the Total OUD counts can be found in section S3.1 Yearly Total OUD counts and Table S1 of the supplemental material. We have added a note in the text and Table 1 caption, to direct the reader to those parts of the supplemental material for more details on the Total OUD counts.

Comment 10.

Table 3. Not sure why for function f(t) it shows beta? Is this a typo?

The Tables can show the units of the parameters.

Response 10.

As noted in the manuscript, f(t) represents probability estimates (proportions) of fatal overdose, therefore we are using Beta(α,β) distribution to represent their respective distributions. The parameters a and b of the Beta distribution are calculated following the formulas presented in the supplemental material with the details of the Empirical Calibration:

$$\alpha = (\frac{1-\mu}{\sigma^2} - \frac{1}{\mu}) \times \mu^2 \quad \text{and} \quad \beta = \alpha \left(\frac{1}{\mu} - 1\right)$$

where μ and σ are estimates of the respective proportions and their dispersity from available data.

The Tables include the necessary information for clear presentation of the relevant information. Please note that most of the values presented in Tables in this text represent counts (i.e., number of individuals or events), probabilities, distributional parameters.

Comment 11.

A brief explanation of the capture-recapture analysis to estimate total OUD counts should be included in order to have some self-content.

Response 11.

It is known that OUD prevalence is underestimated due to several factors including people with OUD not engaged in some level of care, stigma, and limited sources for collecting relevant information. Therefore OUD prevalence estimates are derived from a study using a capture-recapture analysis, a method common in epidemiological studies aimed at adjusting available estimates of sizes of difficult or impossible to observe populations due to movement and lack of available information.

The relevant reference of the published study provides detailed information about the idea and implementation of the capture-recapture analysis in the OUD setting.

Comment 12.

Calibration method. Please explain why there is a specific order? If the model's output does not satisfy any of the 3 target ranges, then the parameter set is discarded?

Response 12.

Thank you for the comment. The order was decided based on results from a sensitivity analysis indicating which RESPOND parameters have the biggest impact on simulated outcomes. Accepted parameter values need to pass all these checks.

We have revised the manuscript accordingly.

Comment 13.

Page 22. Ther authors mentioned that the algorithm can suggest changes un the structure of the model which is much harder than changing ranges for the parameters. Please explain this better and if there was a structural change when the calibration was implemented.

Response 13.

Thank you for the comment. We have added information to better explain this statement.

Comment 14.

Some more specific analysis/comments about the obtained distributions of the parameters should be added.

Response 14.

We have revised the respective Figures captions by adding more detailed information about the resulting distributions.

Comment 15.

In some way the calibration method uses a Bayesian approach. Please comment on this in the conclusions. For instance, what would happen if a strict Bayesian approach were used with priors similar to the ones obtained in this study? Or with very wide ranges for the priors.

Response 15.

Thank you for the comment. In the text we explain how our method is different from a Bayesian approach. In order to avoid confusion we revised the text to avoid terminology related to a Bayesian approach (e.g., “prior” distributions for model parameters, etc.).

Furthermore, as we explain in the Discussion section, the proposed empirical approach can reveal structural issues of the model and the resulting set of accepted parameter values determine a multidimensional space from which randomly selected vectors have higher chances of producing model simulations close to the pre-specified calibration targets. This “focused” multidimensional space can inform the priors of a more comprehensive Bayesian calibration method.

We have revised this section of the manuscript accordingly.

Comment 16.

In the conclusions the authors mentioned that a set of non-identifiable parameters was found. Please mention this particular set to have a better global understanding of the results of this study.

Response 16.

We have revised the manuscript accordingly.

Reviewer 2

This is a very interesting manuscript discussing the calibration of an opioid use disorder model. It is important as it is an area in which data can be limited and modeling can fill a very important need. Calibration can provide more accurate modeling forecasts which are important for decision makers.
While I think the analysis is important, I find that there are some major limitations to this calibration which need to be addressed (or at least detailed within the limitations and explained).

Introduction:

Comment 17.

The focus of the introduction seems to be the importance of modeling and the public health emergency that is opioid use disorder. I suggest focusing more on the importance specifically of calibrating models, and the difference between manual and empiric calibration.

Response 17.

Thank you for the note. We have expanded the "Introduction" section of the manuscript accordingly.

Comment 18.

Paragraph 2 needs citations.

Response 18.

Thank you for the note. We have revised the manuscript accordingly.

Methods:

Comment 19.

The major weakness within this analysis is that the model was only calibrated to 3 years of data, and that data comes from 2013-2015. Limiting data to only 3 years can make it difficult to see trends and calibrate to true trends, and may be the reason that the calibration struggled to meet targets. Why was the decision made to stop here? I assume it is a data limitation. Regardless, this needs to be explained within the manuscript methods AND listed within the limitations. Similarly, there needs to be a defense of using a model calibrated to data from a decade ago to forecast the future, especially within an area where things are rapidly changing (ie laws on drug use and possession, increasing use of fentanyl, changes in MOUD).

Response 19.

This is a very good note. Yes, this is a study limitation and we have added a note to the text. When we implemented this first calibration of the RESPOND model we only had access to the data for those 3 years.

Regarding model projections we note in the manuscript that: "*The calibrated RESPOND model can be used for projections outside the prediction period. However, as in any predictive model, projections are subject to additional uncertainty and based on the strong assumption that the trends fitted with the calibrated model remain unchanged outside the calibration data and period. Model projections should always be used with caution, especially within an area where things are rapidly changing such as the OUD epidemic (changes in laws on drug use and possession, increasing use of fentanyl, changes in MOUD, etc.).*"

Comment 20.

Is relapse estimated to be the same no matter how long someone has been in inactive drug use (ie is my risk of relapsing the same when I last used opioids 1 month ago vs 5 years ago)? Either way, this needs to be explained. The same question with treatment end- am I as likely to leave treatment after 1 month as after 5 years?

Response 20.

As we explain in the manuscript, RESPOND is a Markov State transition model, namely it follows the key assumption that (weekly) transitions depend directly only on the previous state. Rates of transition from inactive to active drug use at a population level are stratified by whether the group in question is not in treatment (versus either treatment or post-treatment). Additionally, transition rates between treatment states are time-varying by calendar year.

Comment 21.

Why were the calibration targets chosen? There is some brief discussion of this, but not specific reasoning for each of the three targets. Why was OUD number chosen when you state it is unobservable AND estimates for 2013-2014 aren't comparable to 2015? Why were detox admissions chosen?

Response 21.

In addition to the reasons we have already discussed in the manuscript about this choice, these targets are also very common across simulation models used for decision making.

We have revised the manuscript accordingly.

Comment 22.

Please further describe methods for internal validation. If this is in the supplement, point to it in the manuscript.

Response 22.

Thank you for the comment. By "internal calibration" is used to describe extended checks to ascertain the accuracy and of different pieces of the code and consistency of derived parameters with relevant information. We have removed this sentence from the text to avoid confusion with the traditional definition of internal validation of a predictive model.

Comment 23.

"To validate externally, we compared model outcomes to data obtained directly from data sources or published studies that were not used in the model calibration process, including deaths due to competing risks, age-sex stratified total OUD and overdose counts, and total OUD counts for the entire simulation period (2013-2015)." Isn't total OUD counts for the simulation period a calibration target? How can it also be an external validation target?

Response 23.

Thank you for the comment. The term "external" validation is to indicate the fact that those data, although referring to the same years, are from other sources and have not been used in our empirical model calibration process.

Discussion:

Comment 24.
Limitations need to be expanded to incorporate some of the issues mentioned above.

Response 24.

Thank you. We have revised the manuscript accordingly.
