# Supplementary material for: Empirical calibration of a simulation model of opioid use disorder
Source: PLoS One. 2025 Mar 27;20(3):e0310763. doi: 10.1371/journal.pone.0310763 (PMC11949371; doi:10.1371/journal.pone.0310763)
Supplement: Attachment — Submitted filename: PONE-D-22-34179_Response to reviewers 20240327.docx [file pone.0310763.s004.docx]

**Empirical Calibration manuscript – PLOS ONE response document**

**We thank the reviewers for the thoughtful comments and suggestions which have considerably helped in improving the structure and the content presented in this manuscript. We have revised the manuscript accordingly and re-submit it for publication at the PLOS ONE journal.**

**In this document we provide a detailed description of how we addressed all comments received by the reviewers.**

1. Please ensure that your manuscript meets PLOS ONE's style requirements, including those for file naming. The PLOS ONE style templates can be found at

[https://journals.plos.org/plosone/s/file?id=wjVg/PLOSOne_formatting_sample_main_body.pdf](https://protect-us.mimecast.com/s/qrI3Cwp5Z4C4LvXNUKLGAI?domain=journals.plos.org)

and

[https://journals.plos.org/plosone/s/file?id=ba62/PLOSOne_formatting_sample_title_authors_affiliations.pdf](https://protect-us.mimecast.com/s/Y7chCxk594cgJLKXURNa26?domain=journals.plos.org).

**Thank you for the comment. We have formatted the manuscript following the PLOSOne guidelines.**

2. Please note that PLOS ONE has specific guidelines on code sharing for submissions in which author-generated code underpins the findings in the manuscript. In these cases, all author-generated code must be made available without restrictions upon publication of the work. Please review our guidelines at [https://journals.plos.org/plosone/s/materials-and-software-sharing#loc-sharing-code](https://protect-us.mimecast.com/s/LhLsCyP5MgfQN7E4UASzor?domain=journals.plos.org) and ensure that your code is shared in a way that follows best practice and facilitates reproducibility and reuse.

**Thank you for the comment.**

**We published the code of the RESPOND simulation here: https://github.com/SyndemicsLab/RESPONDv1**

We added to the **methods section, Page 7**

**The simulation code is available at https://github.com/SyndemicsLab/RESPONDv1.**

3. We note that the grant information you provided in the ‘Funding Information’ and ‘Financial Disclosure’ sections do not match. When you resubmit, please ensure that you provide the correct grant numbers for the awards you received for your study in the ‘Funding Information’ section.

**Thank you. We updated the funding citations for resubmission. The grants that we cite in the resubmission cover letter are correct.**

4. Thank you for stating the following financial disclosure:

“This work was funded by the National Institute on Drug Abuse (R01DA046527) (P30040500) and the National Institute of General Medical Sciences (R35GM141821).”

Please state what role the funders took in the study.  If the funders had no role, please state: ""The funders had no role in study design, data collection and analysis, decision to publish, or preparation of the manuscript.""

If this statement is not correct you must amend it as needed.

Please include this amended Role of Funder statement in your cover letter; we will change the online submission form on your behalf.

**Thank you. We have provided the funding statement in the resubmission cover letter.**

5. Thank you for stating the following in the Acknowledgments/ Funding Section of your manuscript:

“This work was supported by the National Institute on Drug Abuse (R01DA046527, P30DA040500) and the National Institute of General Medical Sciences (R35GM141821).”

We note that you have provided funding information that is not currently declared in your Funding Statement. However, funding information should not appear in the Acknowledgments section or other areas of your manuscript. We will only publish funding information present in the Funding Statement section of the online submission form.

Please remove any funding-related text from the manuscript and let us know how you would like to update your Funding Statement. Currently, your Funding Statement reads as follows:

“This work was funded by the National Institute on Drug Abuse (R01DA046527) (P30040500) and the National Institute of General Medical Sciences (R35GM141821).”

Please include your amended statements within your cover letter; we will change the online submission form on your behalf.

**Thank you.** **We added the funding statement to the cover letter and removed it from the manuscript.**

6. In your Data Availability statement, you have not specified where the minimal data set underlying the results described in your manuscript can be found. PLOS defines a study's minimal data set as the underlying data used to reach the conclusions drawn in the manuscript and any additional data required to replicate the reported study findings in their entirety. All PLOS journals require that the minimal data set be made fully available. For more information about our data policy, please see [http://journals.plos.org/plosone/s/data-availability](https://protect-us.mimecast.com/s/PVwtCzp59jCyRGvrCBP1bh?domain=journals.plos.org).

Upon re-submitting your revised manuscript, please upload your study’s minimal underlying data set as either Supporting Information files or to a stable, public repository and include the relevant URLs, DOIs, or accession numbers within your revised cover letter. For a list of acceptable repositories, please see [http://journals.plos.org/plosone/s/data-availability#loc-recommended-repositories](https://protect-us.mimecast.com/s/dQxZCADoAkSP9ZMRsOAxGF?domain=journals.plos.org). Any potentially identifying patient information must be fully anonymized.

Important: If there are ethical or legal restrictions to sharing your data publicly, please explain these restrictions in detail. Please see our guidelines for more information on what we consider unacceptable restrictions to publicly sharing data: [http://journals.plos.org/plosone/s/data-availability#loc-unacceptable-data-access-restrictions](https://protect-us.mimecast.com/s/YkEZCBBpQ0CkVD3nf7G1TB?domain=journals.plos.org). Note that it is not acceptable for the authors to be the sole named individuals responsible for ensuring data access.

We will update your Data Availability statement to reflect the information you provide in your cover letter.

**We have added the dataset needed to re-create manuscript figures and findings as Supporting Information files.**

7. We note that you have indicated that data from this study are available upon request. PLOS only allows data to be available upon request if there are legal or ethical restrictions on sharing data publicly. For more information on unacceptable data access restrictions, please see [http://journals.plos.org/plosone/s/data-availability#loc-unacceptable-data-access-restrictions](https://protect-us.mimecast.com/s/YkEZCBBpQ0CkVD3nf7G1TB?domain=journals.plos.org).

In your revised cover letter, please address the following prompts:

a) If there are ethical or legal restrictions on sharing a de-identified data set, please explain them in detail (e.g., data contain potentially sensitive information, data are owned by a third-party organization, etc.) and who has imposed them (e.g., an ethics committee). Please also provide contact information for a data access committee, ethics committee, or other institutional body to which data requests may be sent.

b) If there are no restrictions, please upload the minimal anonymized data set necessary to replicate your study findings as either Supporting Information files or to a stable, public repository and provide us with the relevant URLs, DOIs, or accession numbers. For a list of acceptable repositories, please see [http://journals.plos.org/plosone/s/data-availability#loc-recommended-repositories](https://protect-us.mimecast.com/s/dQxZCADoAkSP9ZMRsOAxGF?domain=journals.plos.org).

We will update your Data Availability statement on your behalf to reflect the information you provide.

**Thank you. Please see comment above.**

8. Please review your reference list to ensure that it is complete and correct. If you have cited papers that have been retracted, please include the rationale for doing so in the manuscript text, or remove these references and replace them with relevant current references. Any changes to the reference list should be mentioned in the rebuttal letter that accompanies your revised manuscript. If you need to cite a retracted article, indicate the article’s retracted status in the References list and also include a citation and full reference for the retraction notice.

[Note: HTML markup is below. Please do not edit.]

**We have reviewed the full reference list and did not find any retracted citations. The first citation in the reference list was no longer available at the url listed. [Interim report of the White House Commission on Combating Drug Addiction and the Opioid Crisis. July 31, 2017. Available at:** [**https://www.whitehouse.gov/sites/whitehouse.gov/files/ondcp/commission-interim-report.pdf**](https://www.whitehouse.gov/sites/whitehouse.gov/files/ondcp/commission-interim-report.pdf)**. Accessed August 24, 2017.] This citation has been removed and replaced with: Understanding the Opioid Overdose Epidemic | Opioids | CDC. Published October 7, 2022. Accessed December 29, 2022. https://www.cdc.gov/opioids/basics/epidemic.html**

~~~

Reviewer's Responses to Questions

**Comments to the Author**

Reviewer #1: The manuscript “Empirical Calibration of a Simulation Model of Opioid Use Disorder” is a commendable effort that attempted to calibrate and validate a dynamic, cohort-based Markov model for opioid use disorder in the state of Massachusetts. It is insightful and I have to admit it took me several reads to grasp the model and the calibration approach. I do have a few comments, which I think would help contextualize the findings.

1. The calibration is motivated by the fact that a well-calibrated model could be used to inform policy decisions and comparison of different public health interventions. However, nowhere in the discussion (or introduction) do the authors mention that how opioid epidemic has constantly been evolving and how reasonably recent data would be needed to keep apace with the epidemic. In an ideal world, a predictive validation of the calibrated model using, say, 2016/2017 data would be more informative, but I admit that would be beyond the scope of this paper. Therefore, I would recommend the authors to at least briefly talk about the evolving nature of opioid crisis and how this model fits in that context (the model has time-varying multipliers but can they be extrapolated to the recent years)?

We added the following text to the discussion section, Page 31

**The opioid epidemic and overdose crisis are constantly evolving, and it is impossible to predict future developments. For example, the introduction of synthetic opioids, like fentanyl, drove a major upward inflection in overdose rates around the U.S. that could not have been predicted by any simulation model prior to that event occurring. Similarly, future changes in the supply or substances, or substance use behaviors and preferences could impact the accuracy of projections.** **The calibrated RESPOND model can be used for projections outside the prediction period. However, as in any predictive model, projections are subject to additional uncertainty and they are based on the strong assumption that the trends fitted with the calibrated model remain unchanged outside the calibration data and period.**

2. Related to the point 1), COVID-19 has resulted in some disruption in treatment for OUD. This paper by Huskamp et al. ([https://jamanetwork.com/journals/jama/article-abstract/2774039](https://protect-us.mimecast.com/s/2cyoCERvGoCzWlKqUKyQiZ?domain=jamanetwork.com)) reports that the initiation of OUD medications was lower compared to pre-pandemic, which could affect the transition probabilities for treatment in the model and could result in miscalibration. Likewise, several recent studies have been published that report that there is some disruption in OUD treatment (particularly initiation) which is also likely different for buprenorphine and methadone ([https://www.ncbi.nlm.nih.gov/pmc/articles/PMC8546915/](https://protect-us.mimecast.com/s/ZymBCG6xgqCPJBo8sBia9R?domain=ncbi.nlm.nih.gov),[https://www.sciencedirect.com/science/article/pii/S0955395921004552](https://protect-us.mimecast.com/s/SiLhCJ6AlwC28BPnS8Bmd9?domain=sciencedirect.com), [https://jamanetwork.com/journals/jamanetworkopen/article-abstract/2794637](https://protect-us.mimecast.com/s/klc4CKrBmxCYqD01iXxBtN?domain=jamanetwork.com), [https://jamanetwork.com/journals/jamanetworkopen/article-abstract/2781940](https://protect-us.mimecast.com/s/tPTLCL9DnyugPk53UxkRND?domain=jamanetwork.com), [https://link.springer.com/article/10.1007/s11606-021-07083-y](https://protect-us.mimecast.com/s/0rGmCM8gozCL52GJT7HglG?domain=link.springer.com)). I think this is important because the model may likely be miscalibrated in the current time when they are needed the most. I would recommend the authors to discuss briefly how they see the model and the calibration process would fare going forward.

We added the following to the discussion, page 31

**Similarly, the COVID-19 pandemic likely interrupted OUD treatment and drug use patterns and resulted in discontinuity of calibrated trends. As data become available, however, this method makes it possible to rapidly incorporate COVID-era trends into the simulation.**

3. The authors mention that they could not use total OUD counts from 2013 and 2014 as calibration targets because it was not comprehensive enough. But the capture-recapture analysis paper they cite mentions that a new dataset (MATRIS) was added to MA PHD in 2013 and the paper stratified the analysis by 2011-2012 and 2013-2015. I found it a bit inconsistent, so looking for some clarification here. You don’t need to add anything in the manuscript if I am misunderstanding and you could just explain here.

**The reviewer is correct that in our prevalence estimation work using a capture recapture approach, we found that the MATRIS database, which tracks EMS calls and ambulance administrations of naloxone, was critically important to prevalence estimates and that any estimate that did not use MATRIS was likely less accurate. Because MATRIS did not come “on line” in the database until 2013, we did not want to “fit” the simulation to the “pre-MATRIS” estimates.**

6. PLOS authors have the option to publish the peer review history of their). If published, this will include your full peer review and any attached files.


If you choose “no”, your identity will remain anonymous but your review may still be made public.
